# A novel method for expediting the development of patient-reported outcome measures and an evaluation of its performance via simulation

**DOI:** 10.1186/s12874-015-0071-5

**Published:** 2015-09-29

**Authors:** Lili Garrard, Larry R. Price, Marjorie J. Bott, Byron J. Gajewski

**Affiliations:** Department of Biostatistics, University of Kansas Medical Center, Mail Stop 1026, 3901 Rainbow Blvd., Kansas City, KS 66160 USA; College of Education, Texas State University, San Marcos, TX 78666 USA; University of Kansas School of Nursing, Mail Stop 4043, 3901 Rainbow Blvd., Kansas City, KS 66160 USA

**Keywords:** OBID, Bayesian psychometrics, Ordinal data analysis, Bayesian IRT, Patient-reported outcome measures, PROMs

## Abstract

**Background:**

Developing valid and reliable patient-reported outcome measures (PROMs) is a critical step in promoting patient-centered health care, a national priority in the U.S. Small populations or rare diseases often pose difficulties in developing PROMs using traditional methods due to small samples.

**Methods:**

To overcome the small sample size challenge while maintaining psychometric soundness, we propose an innovative Ordinal Bayesian Instrument Development (OBID) method that seamlessly integrates expert and participant data in a Bayesian item response theory (IRT) with a probit link model framework. Prior distributions obtained from expert data are imposed on the IRT model parameters and are updated with participants’ data. The efficiency of OBID is evaluated by comparing its performance to classical instrument development performance using actual and simulation data.

**Results and Discussion:**

The overall performance of OBID (i.e., more reliable parameter estimates, smaller mean squared errors (MSEs) and higher predictive validity) is superior to that of classical approaches when the sample size is small (e.g. less than 100 subjects). Although OBID may exhibit larger bias, it reduces the MSEs by decreasing variances. Results also closely align with recommendations in the current literature that six subject experts will be sufficient for establishing content validity evidence. However, in the presence of highly biased experts, three experts will be adequate.

**Conclusions:**

This study successfully demonstrated that the OBID approach is more efficient than the classical approach when the sample size is small. OBID promises an efficient and reliable method for researchers and clinicians in future PROMs development for small populations or rare diseases.

**Electronic supplementary material:**

The online version of this article (doi:10.1186/s12874-015-0071-5) contains supplementary material, which is available to authorized users.

## Background

The Institute of Medicine (IOM) [1] released a landmark report, *Crossing the Quality Chasm*, which highlighted patient-centered care as one of the six specific aims (the others being safety; effectiveness; timeliness; efficiency; and equity) that defined quality health care. To promote patient-centered care, national entities such as the National Institute of Health (NIH) [2], the U. S. Department of Health and Human Services (DHHS) Food and Drug Administration (FDA) [3], the National Quality Forum (NQF) [4], and the Patient-Centered Outcomes Research Institute (PCORI) [5] have published specific guidelines on the development of patient-reported outcome measures (PROMs). The guidelines unanimously emphasize the critical requirement of rigorous psychometric testing for any new or adapted PROMs that often are designed as survey instruments. PROMs serve a critical role in translational research as data collected using PROMs are commonly used as primary or surrogate endpoints for clinical trials and studies in humans, which are essential for promoting both clinical application and public awareness. However, the lengthy process of developing valid and reliable psychometric instruments (e.g., PROMs) is recognized as one of the greater barriers for disseminating and transitioning research findings into clinical practice in a timely manner.

For decades classical instrument development methodologies (e.g., frequentist approach to factor analysis that ignores prior information regarding item reliability) dominated the psychometric literature [6]. Bayesian methods have been severely limited until modern computation techniques provided researchers the capacity to employ Bayesian inference in actual applications [7]. As Bayesian inference becomes more popular, limitations arise with the use of classical (i.e. frequentist) methods when developing instruments or PROMs for small populations (e.g., in cases of rare diseases). Since it is not the intent of the authors to provide a comprehensive review of both classical and Bayesian statistical approaches, we focus our discussions on two co-existing issues with the classical approach to confirmatory factor analysis (CFA) in establishing evidence of construct validity: (a) the requirement of large samples, and (b) modeling ordinal data as continuous.

Two essential components of establishing evidence that scores acquired by an instrument exhibit score validity include content and construct-related evidence [8, 9]. Subject experts’ opinions are typically consulted in evaluating the content of items, such as how well the items match the empirical indicators of the construct(s) of interest, and the relevancy and clarity of the items. The items evolve through rigorous revision (e.g., iteratively through pilot-testing with a small representative sample of respondents) until the instrument is deemed ready for establishing construct validity evidence through a statistical technique such as factor analysis. It is a common practice to conduct expert evaluation for content analysis; however, under the classical setting data collected from the experts are not utilized in establishing construct validity as content validity focuses on the instruments rather than measurements [10]. The expert and participant data are analyzed separately, which results in potential loss of information and leads to the increasing demand for a large participant sample.

There is no consensus among health care researchers regarding the number of subjects required for CFA. Knapp and Brown [11] list several competing rules regarding the number of subjects required and argue that original studies on factor analysis (e.g., [12]) only assumed very large samples relative to the number of items, and made no recommendations on a minimum sample size. Pett et al. [6] make the recommendation of at least 10 to 15 subjects per item, a commonly suggested ratio in psychometric literature. However, Brown [13] urges researchers to not rely on these general rules of thumb and proposes more reliable model-based (e.g., Satorra-Saris’s method) and Monte Carlo methods to determine the most appropriate sample size for obtaining sufficient statistical power and precision of parameter estimates. A recent systematic review study [14] on sample size used to validate newly-developed PROMs reports that 90 % of the reviewed articles had a sample size ≥ 100, whereas 7 % had a sample size ≥ 1000. In addition, Weenink, Braspenning and Wensing [15] explore the potential development of PROMs in primary care using seven generic instruments. The authors report challenges of low response rates to questionnaires (i.e., small sample), and that a replication in larger studies would require a sample size of at least 400 patients.

Apart from the large sample issue, the other issue concerns how data are analyzed using traditional approaches. The most common form of data acquired from measurement instruments in the social, behavioral, and health sciences are ordinal; however, such data often are analyzed without regard for their ordinal nature [7]. The practice of treating ordinal data as continuous is considered a controversy and has generated debates in the psychometric literature [16]. With solid theoretical developments in ordinal data modeling, it is considered best practice to use modeling techniques that treat ordinal data as ordinal. Structure equation modeling (SEM) with categorical variables first was introduced by Muthén [17] in a landmark study that revolutionized psychometric work. Although techniques for handling ordinal data in latent variable analysis have been incorporated into several commercial statistical software (e.g., Mplus) since the 1980’s, it is only in 2012 that the free R package *lavaan* incorporated the weighted least squares means- and variance-adjusted (WLSMV) estimator for performing ordinal CFA during its version 0.5-9 release [18, 19]. Ordinal CFA offers new insight for modeling ordinal data under the classical setting; yet it is still challenged by small samples, as we will show in this study. A more complete solution is needed to resolve both limitations and still provide reliable model estimates.

New methods proposed by Gajewski, Price, Coffland, Boyle and Bott [20] and Jiang, Boyle, Bott, Wick, Yu and Gajewski [21] use Bayesian approaches to resolve the sample size limitation of traditional CFA. The Integrated Analysis of Content and Construct Validity (IACCV) approach establishes a unified model that seamlessly integrates the content and construct validity analyses [20]. *Prior* distributions derived from content subject experts’ data are updated with participants’ data to obtain a *posterior* distribution. Under the IACCV approach, some of the response burden from the participants can be alleviated by using experts; thus fewer participants are needed to achieve the desired validity evidence in developing instruments. Using both simulation data and real data, Bayesian Instrument Development (BID) [21] advances the theoretical work of IACCV by demonstrating the superior performance of BID to that of classical CFA when the sample size is small. BID also advances the practical application of IACCV by incorporating the methodology into a user-friendly GUI software that is shown to be reliable and efficient in a clinical study for developing an instrument to assess symptoms in heart failure patients. Although BID has shown great potential, the method is limited by the assumption of continuous participant response data. As previously mentioned, many clinical questionnaires data are collected as ordinal or binary (a special type of ordinal data). Given this fact, there is an urgent need to adapt the BID approach for ordinal responses.

In this article, we propose an Ordinal Bayesian Instrument Development (OBID) approach within a Bayesian item response theory (IRT) framework to further advance BID methodology for ordinal data. On first glance, the current study appears to be a straightforward extension from previous studies; however it differs from previous studies and contributes to the literature from several perspectives. First, as previously mentioned, ordinal or binary data are the most common form of data collected using clinical instruments. The underlying distribution assumption required by continuous data modeling is often violated due to skewed responses. Our study effectively promotes the proper usage of ordinal data modeling methods and brings awareness to a broader audience regarding the psychometric integrity of the measurement, which is essential for the development of PROMs and clinical trial outcomes. Although several simulation studies on Bayesian IRT models have been discussed in the literature, the studies arbitrarily select non-informative or weakly informative priors for model parameters without a clear elicitation process (e.g., [22, 23]). Alternatively, our approach is distinct because we leverage experts in elicitation of the priors for the IRT parameters. Second, the consideration of the predictive validity of the instrument [9] that is often neglected in the literature is addressed here. These important steps are implemented in the simulation study for contribution to the methodological literature.

Results from our approach also have several practical implications to the development of PROMs, as OBID overcomes the small sample size (e.g., patients from small populations) challenge while maintaining psychometric integrity. Special considerations for reducing the resource and cost burden incurred by researchers and clinicians are provided through the usage of fast and reliable free R packages to implement the OBID methodology. In our approach, a Markov chain Monte Carlo (MCMC) procedure is implemented to estimate the model parameters; we provide general guidelines for selecting tuning parameters required in the MCMC procedure for achieving appropriate acceptance/rejection rates. Our proposed method demonstrates that the overall performance of OBID (i.e., more reliable parameter estimates, smaller mean squared errors (MSE) and higher predictive validity) is superior to that of ordinal CFA when the sample size is small. Most importantly, OBID promises an efficient and reliable method for researchers and clinicians in future PROM development.

## Methods

OBID further advances the work of Jiang et al. [21] that expands IACCV of Gajewski et al. [20], by adapting the BID methodology for ordinal scale data. Here we demonstrate the OBID approach using a unidimensional (i.e., single factor) psychometric model and refer interested readers to Gajewski *et al.* and Jiang *et al.* for a detailed description of the general model and the BID approach. In addition, we use a similar model and incorporate mathematical notation as presented in Jiang *et al.* to maintain some level of consistency between both studies.

### Bayesian IRT model

Prior to introducing the OBID model, it is important to clarify that both OBID and BID are CFA-based approaches. IRT is a psychometric technique that provides a *probabilistic* framework for estimating how examinees will perform on a set of items based on their ability and characteristics of the items [24]. IRT is a model-based theory of statistical estimation that conveniently places persons and items on the same metric based on the probability of response outcomes. Traditional factor analysis is based on a *deterministic* model and does not rest on a probabilistic framework. Here we provide a probabilistic connection between our approach and IRT, by using Bayesian CFA, including an inherently probabilistic framework. From a modeling perspective, IRT is the ordinal version of traditional factor analysis. When all manifest variables are ordinal, the traditional factor analysis model is equivalent to a two-parameter IRT model with a probit link function [7, 25]. The two-parameter IRT model with the probit link can be written as

1 $$ {y}_{ij}=c\ \mathrm{if}\ {y}_{ij}^{*}\in \left({T}_{j\left(c-1\right),}{T}_{jc}\right];\ i=1,\dots,\ N,\ j=1, \dots,\ P,\ c=1,\dots,\ {C}_j $$

2 $$ {y}_{ij}^{*}={\alpha}_j+{\lambda}_j{f}_i+{\varepsilon}_{ij};\ {f}_i \sim N\left(0,\ 1\right),\ {\varepsilon}_{ij} \sim N\left(0,\ 1\right),\ i=1,\dots,\ N,\ j=1, \dots,\ P, $$

where ${y}_{ij}$ is the *i*th participant’s ordinal response to the *j*th item; and ${C}_j$ is the total number of response categories for item *j* (e.g., a five-point Likert scale). The ordinal response ${y}_{ij}$ is linked to ${y}_{ij}^{*}$, an underlying continuous latent variable that follows a normal distribution, through a set of ${C}_j-1$ ordered cut-points, ${T}_{jc}$, on ${y}_{ij}^{*}$. The probability of a subject selecting a particular response category is indicated by the probability that ${y}_{ij}^{*}$ falls within an interval defined by the cut-points ${T}_{jc}$. In IRT, the continuous latent variable ${y}_{ij}^{*}$ is characterized by two item-specific parameters: ${\alpha}_j$, the negative difficulty parameter for the *j*th item and ${\lambda}_j$, the discrimination parameter for item *j.* In addition, the underlying latent ability ${f}_i$ of the subjects is constrained to follow a standard normal and ${\varepsilon}_{ij}$ is the measurement error [7].

To see the equivalence between the IRT model and traditional factor analysis model, note that a classical unidimensional factor analysis model can be expressed as

3 $$ {z}_{ij}^{*}={\rho}_j{f}_i+{e}_{ij};\ i=1,\dots,\ N,\ j=1,\dots,\ P, $$

where ${z}_{ij}^{*}$ represents the standardized ${y}_{ij}^{*}$ from equations 1 and 2; ${f}_i$ is the *i*th participant’s factor score for the domain; ${\rho}_j$ is the factor loading or item-to-domain correlation for the *j*th item; and ${e}_{ij}$ represents the measurement errors or sometimes referred to as latent unique factors or residuals. ${f}_i$ is assumed to follow a standard normal distribution, which implies that ${e}_{ij}\sim N\left(0,\;1-{\rho}_j^2\right)$ where ${\rho}_j^2$ is the reliability of the *j*th item. The standardization of ${y}_{ij}^{*}$ is expressed by

4 $$ \frac{y_{ij}^{*}-{\alpha}_j}{\sqrt{1+{\lambda}_j^2}}=\frac{\lambda_j}{\sqrt{1+{\lambda}_j^2}}{f}_i+\frac{\varepsilon_{ij}}{\sqrt{1+{\lambda}_j^2}};\ i=1,\dots,\ N,\ j=1,\dots,\ P, $$

such that the IRT model parameter ${\lambda}_j$ can be interpreted interchangeably through the item-to-domain correlations ${\rho}_j$ using the following expressions

5 $$ {\lambda}_j=\frac{\rho_j}{\sqrt{1-{\rho}_j^2}} $$

6 $$ {\rho}_j=\frac{\lambda_j}{\sqrt{1+{\lambda}_j^2}}. $$

Equations 5 and 6 can be interpreted such that an item that well-discriminates among individuals with different abilities also will have a high item-to-domain correlation. The true Bayesian application comes from specifying appropriate prior distributions on the IRT parameters, which leads us into the essence of the OBID method.

### OBID – expert data and model

Eliciting subject experts’ perception regarding the relevancy of each item to the domain (construct) of interest is a common practice to aid in verifying content validity evidence. For example, during instrument development, a logical structure is developed and applied in a way that maps the items on the test to a content domain [8]. In this way, the relevance of each item and the adequacy with which the set of items represents the content domain is established. To illustrate, a panel of subject experts are asked to review a set of potential items and instructed to provide response for questions such as “please rate the relevancy of each item to the overall topic of [domain].” The response options are generally designed on a four-point Likert scale that ranges from “not relevant” to “highly relevant.” Gajewski, Coffland, Boyle, Bott, Price, Leopold and Dunton [26] laid important groundwork from an empirical perspective by demonstrating the approximate equivalency of measuring content validity using relevance scales versus using correlation scales. In other words, content validity oriented evidence can be statistically interpreted as a representation of the experts’ perceptions regarding the item-to-domain latent correlation [21].

Continuing the notations from Jiang *et al.*, suppose the expert data are collected from a panel of $k=1,\dots,\;K$ experts that respond to $j=1, \dots,\;P$ items. Let ***X*** denote the *K* × *P* matrix of observed ordinal responses where the *x*_*jk*_th entry represents the *k*th expert’s opinion regarding the relevancy of the *j*th item to its assigned domain. Similarly, the *k*th expert’s latent correlation between the *j*th item and its respective domain is denoted by *ρ*_*jk*_ and is related to *x*_*jk*_ using the following function, with correlation cut-points suggested by Cohen [27]:

7 $$ {x}_{jk}=\left\{\begin{array}{ll}1\ "\mathrm{not}\ \mathrm{relevant}"\hfill & \mathrm{if}\ 0.00\le {\rho}_{jk}<0.10\hfill \\ {}2\ "\mathrm{somewhat}\ \mathrm{relevant}"\hfill & \mathrm{if}\ 0.10\le {\rho}_{jk}<0.30\hfill \\ {}\begin{array}{l}3\ "\mathrm{quite}\ \mathrm{relevant}"\hfill \\ {}4\ "\mathrm{highly}\ \mathrm{relevant}"\hfill \end{array}\hfill & \begin{array}{l}\mathrm{if}\ 0.30\le {\rho}_{jk}<0.50\hfill \\ {}\mathrm{if}\ 0.50\le {\rho}_{jk}\le 1.00\hfill \end{array}\hfill \end{array}\right\}. $$

A sensitivity analysis conducted by Gajewski et al. [26] demonstrated the approximate equivalency of using correlation scale and using relevancy scale to measure content validity, under both equally-spaced (i.e., $0.00\le {\rho}_{jk}<0.25$, $0.25\le {\rho}_{jk}<0.50$, $0.50\le {\rho}_{jk}<0.75$, and $0.75\le {\rho}_{jk}<1.00$) and unequally spaced (i.e., equation 2) cut-points assumptions. One of the reviewers pointed out that under certain circumstances, the equally-spaced transformation might be more appropriate (e.g., a panel with moderate level of expertise in the area of interest) [26]. However, the results were based on unexpected secondary findings, which require further confirmation in a more thorough study [26]. For the purpose of the current study, we want to primarily focus on showcasing a proper method of establishing evidence for construct validity using carefully selected “true” subject experts. For developing PROMs, the level of expertise of the selected subject experts’ has a direct impact on the validity of the measurement instrument.

In our assumed single factor model, the item-to-domain correlation based on pooled information from all experts can be denoted by ${\rho}_j= corr\left(f,{z}_j\right)$, where $f$ represents the domain factor score and is typically assumed to follow a standard normal distribution; and ${z}_j$ represents the standardized response of item *j*. To ensure the proper range of correlations, Fisher’s transformation is used to transform ${\rho}_j$ and we denote ${\mu}_j$ as

8 $$ {\mu}_j=g\left({\rho}_j\right)=\frac{1}{2} log\frac{1+{\rho}_j}{1-{\rho}_j}. $$

A hierarchical model that combines all experts and includes all items is defined by

9 $$ g\left({\rho}_{jk}\right)=g\left({\rho}_j\right)+{e}_{jk}, $$

where ${e}_{jk}\sim N\left(0,\;{\sigma}^2\right)$. Following the BID model, the prior distribution of the experts after Fisher’s transformation is approximately normal and can be expressed by

10 $$ {\mu}_j=g\left({\rho}_j\right) \sim N\left(g\left({\rho}_{0j}\right),\ \frac{1}{n_{0j}}\right), $$

where $g\left({\rho}_{0j}\right)$ is the transformed prior mean item-to-domain correlation; and ${n}_{0j}=5\times K$ is the prior samples size such that each expert is equivalent to approximately five participants [21]. This approximation is based on a weighted average from previous study findings by Gajewski et al. [20, 26] and Jiang et al. [21]. The prior sample size ${n}_{0j}$ can be approximated by computing the ratio of the variance of the subject experts’ transformed ${\rho}_j$ and the variance of the participants’ transformed ${\rho}_j$ (i.e., using a flat prior). The “five participants” assumption will be further evaluated as more data become available. Moreover, the current approximation is solely needed to help execute the simulation study and not used within any real data application.

Informative priors only should be used when appropriate content information is available. When items are substantially revised without further review from subject experts, flat priors should be used. Although eliciting prior distribution from subject experts is highlighted, we are not restricted solely to this approach. When reliable and relevant external data are available (i.e., not necessarily experts), a different data driven approach can be utilized. For instance, developing PROMs for pediatric populations can be challenging due to low disease incidence in children, thus resulting in small samples. Reliable evidence from the adult populations can be treated as a “general prior” for establishing construct validity in the pediatric populations.

### OBID – participant data and model

Establishing evidence of score validity involves integrating various strategies or techniques culminating in a comprehensive account for the degree to which existing evidence and theory support the intended interpretation of scores acquired from the instrument [24]. From a purely psychometric or statistical perspective, establishing content validity evidence has traditionally been carried out separately from establishing evidence of construct validity. Importantly, the OBID approach more closely aligns with current practice forwarded by the American Educational Research Association (AERA), American Psychological Association (APA) and the National Council on Measurement in Education (NCME) [8] regarding an integrated approach to establishing evidence for score validity in relation to practical use. OBID seamlessly integrates content and construct validity analyses into a single process, which alleviates the need for a large participant sample. The previously introduced IRT with a probit link model, expressed by equations 1 and 2, is used to model the ordinal participant responses. The likelihood for ${y}_{ij}^{*}$ is

11 $$ L\left({\boldsymbol{y}}^{*}\Big|\boldsymbol{\alpha}, \boldsymbol{\lambda}, \boldsymbol{f}\right)={\displaystyle {\prod}_{i=1}^N{\displaystyle {\prod}_{j=1}^PN\left({y}_{ij}^{*}\Big|{\alpha}_j+{\lambda}_j{f}_i,\ 1\right)}}. $$

By equations 5, 8 and 10 and the delta method, we specify the prior distribution of the item discrimination parameter ${\lambda}_j$ through a normal approximation where

12 $$ {\lambda}_j \sim N\left(\frac{ \exp \left(2{\mu}_j\right)-1}{2 \exp \left({\mu}_j\right)},\frac{{\left\{ \exp \left(2{\mu}_j\right)+1\right\}}^2}{4{n}_{0j} \exp \left(2{\mu}_j\right)}\right). $$

Since the item-to-domain correlation ${\rho}_j$ does not depend on the negative item difficulty parameter ${\alpha}_j$, we assign the prior ${\alpha}_j\sim N\left(0,\;1\right)$ according to recommendations made by Johnson and Albert [7]. The full posterior distribution is

$$ \pi \left(\boldsymbol{\alpha}, \boldsymbol{\lambda} \Big|{\boldsymbol{y}}^{*},\boldsymbol{f}\right)={\displaystyle \prod_{i=1}^N}{\displaystyle \prod_{j=1}^P}N\left({y}_{ij}^{*}\Big|{\alpha}_j+{\lambda}_j{f}_i,\ 1\right)\times {\displaystyle {\prod}_{i=1}^NN}\left({f}_i\Big|0,\ 1\right)\times {\displaystyle {\prod}_{j=1}^PN}\left({\alpha}_j\Big|0,\ 1\right) $$

13 $$ \times {\displaystyle \prod_{j=1}^P}N\left({\lambda}_j\left|\frac{ \exp \left(2{\mu}_j\right)-1}{2 \exp \left({\mu}_j\right)},\ \frac{{\left\{ \exp \left(2{\mu}_j\right)+1\right\}}^2}{4{n}_{0j} \exp \left(2{\mu}_j\right)}\right.\right)\times {\displaystyle {\prod}_{j=1}^PN}\left({\mu}_j\left|{\mu}_{0j},\ \frac{1}{n_{0j}}\right.\right). $$

### OBID model estimation

The integration of content and construct validity analyses requires us to calculate the posterior distribution of the expert data and use the posterior inferences as priors for the participant model parameters, as expressed in equation 13. Prior to eliciting expert opinions, it is natural to assume that no information exists regarding the items. Thus, flat or non-informative priors can be specified in equations 9 and 10 such that ${\sigma}^2\sim IG\left(0.00001,\;0.00001\right)$ and ${\mu}_j=g\left({\rho}_j\right)\sim N\left(0,\;3\right)$. The MCMC procedure is implemented in the free software WinBUGS [28] to estimate the posterior distribution of ${\lambda}_j$ based on ${\mu}_j$ from the experts’ data. Three chains are used with a burn-in sample of 2000 draws. The next 10,000 iterations are used to calculate the posterior inferences that form the priors of ${\lambda}_j$ in the participant IRT model.

The estimation of ${\lambda}_j$’s in the participant model can be obtained by using the *MCMCordfactanal* function included in the free R package *MCMCpack* [29]. To be specific, the R function utilizes a Metropolis-Hastings within Gibbs sampling algorithm proposed by Cowles [30]. Similarly, the posterior estimation of ${\lambda}_j$’s is based on 10,000 iterations after 2000 burn-in draws. The item-to-domain correlations ${\rho}_j$’s can be subsequently calculated from the estimated ${\lambda}_j$’s via equation 6. An important consideration in any MCMC procedure is the choice of a tuning parameter that influences the appropriate acceptance or rejection rate for each model parameter. According to Gelman, Carlin, Stern and Rubin [31] and Quinn [25], the proportion of accepted candidate values should fall between 20- 50 %. There is no standard “formula” for selecting the most appropriate tuning parameter. As Quinn suggested, users typically adjust the value of the turning parameter through trial and error. In the upcoming discussion of the simulation study, we have found that the following tuning parameter values 1.00, 0.70, 0.50, and 0.30 appear to work well for sample sizes 50, 100, 200, and 500, respectively.

### Predictive validity

An essential yet often neglected instrument evaluation step is the assessment of predictive validity. Predictive validity is sometimes referred to as criterion-related validity whereas the criterion is external to the current predictor instrument. From a statistical standpoint, assuming the availability of an appropriate criterion, the predictive validity is directly indicated by the size of the correlation between predictor scores and criterion scores. However, demonstrating construct validity of an instrument may not always support the establishment of predictive validity due to factors such as range restriction, where the relevant differences on the predictor or criterion are eliminated or minimized. Thus, the performance of predictive validity depends entirely on the extent to which predictor scores correlate with criterion scores intended to be predicted [9, 24].

In this article we compare the OBID predictive validity with that of the traditional approach. Using the test scores or the underlying latent ability parameter ${f}_i$ of the subjects, the validity coefficient is defined as

14 $$ \gamma = corr\left\{E\left(\boldsymbol{f}\right),{\boldsymbol{f}}^T\right\}, $$

where E (***f***) is the posterior mean of the test scores and ***f***^*T*^ represents the set of true test scores. In our simulation study, the criterion is assumed to be perfectly measured; thus the correlation of the test score ${f}_i$ (i.e., the ability parameter) and the criterion score is the same as the validity coefficient corrected for attenuation in the criterion only.

## Results

### Simulation study

In this section, we use simulated data to test the OBID approach by comparing its overall performance to classical instrument development, specifically through the comparison of parameter estimates, MSEs, and predictive validity. Two important assumptions are made by Jiang et al. [21] for BID that also apply to the OBID simulation setting. First, all experts are assumed to agree in regards to interpreting the concept of correlation in their opinions about the items’ relevancy; and second, the experts’ data are assumed to be correlated with the participants’ data with the indication of having either the same opinions or very similar opinions. In addition, the BID study makes the assumption that the true item-to-domain correlation is ${\rho}^T=0.50$ for all items. Upon careful consideration, we have decided against this assumption for the current study as in reality it is rare for all items to have the same moderate item-to-domain correlation. Thus, we employ a mixture of low, moderate, and high (i.e., 0.30, 0.50, and 0.70) true item-to-domain correlations in this simulation study. The simulation is conducted in R software version 3.1.2 [19], including additional inferences and simulation plots. OBID parameter estimation is obtained using the previously introduced *MCMCordfactanal* function in the R package *MCMCpack* [29]*.* In addition, for comparison purposes ordinal CFA is performed using the *cfa* function in the R package *lavaan* version 0.5-17 [18].

Working with the assumed unidimensional model, a five-way factorial design is used to simulate the data. The simulation factors include number of items on the instrument (4, 6, 9) and number of response categories per item (2, 5, 7). For simplicity and demonstration purposes, we assume that all items have the same number of response categories in the current simulation. However, it is possible for items to have different number of response categories on a questionnaire. In addition, we examine the effect of expert bias using different number of participants (50, 100, 200, 500), number of subject experts (2, 3, 6, 16), and types of expert bias (unbiased, moderately biased, highly biased). We define unbiased experts as ${\rho}_0={\rho}^T$, moderately biased experts as ${\rho}_0={\rho}^T+0.1$, and highly biased experts as ${\rho}_0={\rho}^T+\frac{1-{\rho}^T}{2}$. This design results in 432 different combinations of factors. The detailed simulation strategy is as follows:

1. Simulate standardized participant responses ${z}_{ij}^{*}$ and convert to ${y}_{ij}^{*}$ based on the classical factor model (equation 3). The true item-to-domain correlation ${\rho}^T$ is specified as ${\rho}^T=\left(0.50,\;0.30,\;0.70,\;0.50\right)$ for all four item scenarios, ${\rho}^T=\left(0.30,\;0.50,\;0.70,\;0.70,\;0.30,\;0.50\right)$ for all six item scenarios, and ${\rho}^T=\left(0.30,\;0.50,\;0.70,\;0.70,\;0.30,\;0.50,\;0.70,\;0.50,\;0.30\right)$ for all nine item scenarios.
2. Convert ${y}_{ij}^{*}$ to ordinal responses ${y}_{ij}$ using equation 1 and percentile-based cut points. When the number of categories is binary, or $C=2$, the single cut point is the 50^th^ percentile of the standard normal. When the number of categories is polytomous, or $C>2$, the cut points are defined as the $\left(\frac{1}{C}, \dots,\;\frac{C-1}{C}\right)$ th percentile of the standard normal.
3. Define prior for the participant IRT model (equation 2) item discrimination parameter ${\lambda}_j$ using equations 8, 10 and 11. Recall that we previously specify the prior for the negative item difficulty parameter ${\alpha}_j$ as ${\alpha}_j\sim N\left(0,\;1\right)$.
4. Select appropriate tuning parameters to ensure 20-50% acceptance rate. As previously mentioned, we have found through trial and error that the following tuning parameter values 1.00, 0.70, 0.50, and 0.30 appear to work well for sample sizes *N =* 50, 100, 200, and 500, respectively.
5. Fit the IRT model on the simulated datasets created in steps 1–2 via *MCMCpack* and obtain estimates for ${\lambda}_j$ and ${\rho}_j$ using equations 5 and 6.
6. Fit the ordinal CFA model on the same simulated datasets created in steps 1–2 via *lavaan* and estimate ${\rho}_j$.
7. Perform 100 simulations for each of the scenarios defined by the simulation factors.

The simulation process for one type of expert bias takes about two days to run on an Intel Core i7 3.40 GHz computer with 32GB of RAM. In order to compare the overall performances of OBID and CFA, we calculate the average MSE of the item-to-domain correlation estimates and the MSE of the validity coefficient estimates across 100 simulations with 5000 MCMC iterations and 2000 burn-in draws. We denote ${\widehat{\rho}}_j(s)$ as the OBID posterior mean or CFA parameter estimate of the *s*th iteration and ${\overline{\rho}}_j=\frac{\sum_{s=1}^{100}{\widehat{\rho}}_j(s)}{100}$. Then $MSE\left({\widehat{\rho}}_j\right)=\frac{\sum_{s=1}^{100}{\left\{{\widehat{\rho}}_j(s)-{\rho}_j^T\right\}}^2}{100}$ and $\overline{MSE}=\frac{\sum_{j=1}^PMSE\left({\widehat{\rho}}_j\right)}{P}$; ${\left\{ Bias\left({\widehat{\rho}}_j,{\rho}_j^T\right)\right\}}^2={\left({\overline{\rho}}_j-{\rho}_j^T\right)}^2$ and $\overline{Bias^2}=\frac{\sum_{j=1}^P{\left\{ Bias\left({\widehat{\rho}}_j,{\rho}_j^T\right)\right\}}^2}{P}$. For evaluating the predictive validity, we denote $\widehat{\gamma}(s)= corr\left[E\left\{\widehat{f_i}(s)\right\},{f}_i^T(s)\right]$ as the correlation between the posterior mean of estimated factor scores and true factor scores for the *s*th iteration. As previously mentioned, we assume that the true criterion is perfectly measured such that ${\gamma}^T=1$. Then $MSE\left(\gamma \right)=\frac{\sum_{s=1}^{100}{\left\{\widehat{\gamma}(s)-{\gamma}^T\right\}}^2}{100}$. In addition, due to concerns about the performance of CFA with small samples, we record the frequency that ordinal CFA fails to converge and/or produces “bad” estimates such that ${\rho}_j\notin \left[-1,\;1\right]$*.*

**Fig. 1:**
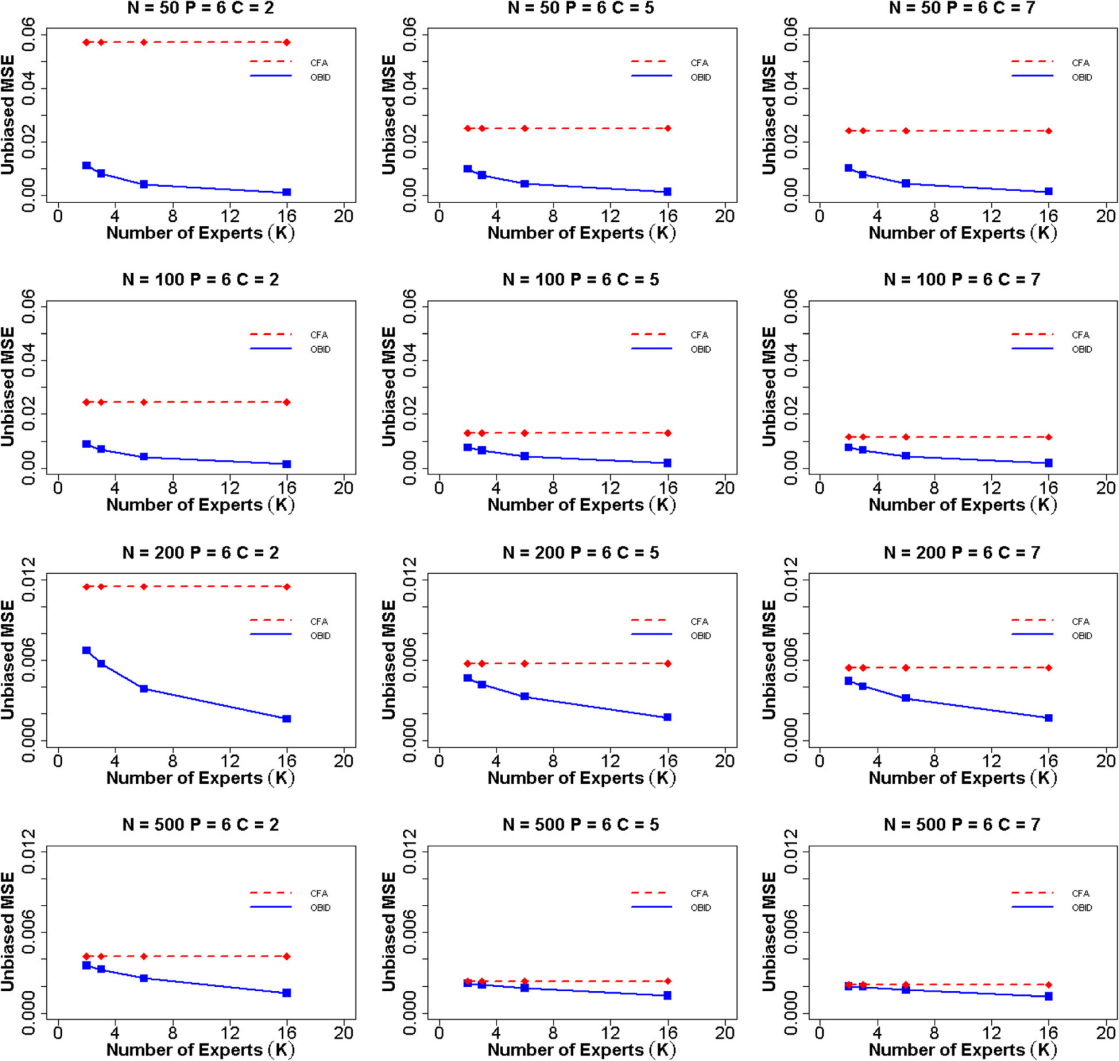
Average MSE of item-to-domain correlation $\rho$ for six items and unbiased experts. Average mean squared error (MSE) for item-to-domain correlation $\rho$ using OBID (solid blue line) and ordinal CFA (dashed red line) when *P = 6* (number of items) and experts are unbiased {${\rho}_0=\left(0.30,\;0.50,\;0.70,\;0.70,\;0.30,\;0.50\right)\Big\}$. The participant sample sizes are *N =* 50, 100, 200, and 500. The numbers of response categories are *C =* 2, 5, and 7, and the numbers of experts are *K =* 2, 3, 6, and 16. *Note*. OBID = Ordinal Bayesian Instrument Development; CFA = Confirmatory Factor Analysis

Figure 1 shows the average MSE of item-to-domain correlation $\rho$ for unbiased experts when the number of items (*P*) is six. The participant sample sizes are *N =* 50, 100, 200, and 500. The numbers of response categories are *C =* 2, 5, and 7, and the numbers of experts are *K =* 2, 3, 6, and 16. The MSE for CFA does not change with the number of experts (dashed line) as the expert content validity information is not utilized under the traditional approach. Thus the prior information has no effect on the CFA estimates across different choices for the number of experts. The OBID MSE (solid line) is consistently smaller than the CFA MSE, regardless of sample size and number of response categories, demonstrating the superior performance of the OBID approach. OBID is most promising for smaller samples (e.g., *N =*50 or 100). In addition, the OBID MSE decreases as the number of experts increases, with the largest reduction occurring approximately between 3–6 experts. When the number of response categories is binary (*C =* 2), we observe the largest vertical distance between the OBID MSE and the CFA MSE. This vertical distance reduces as the number of response categories increase, due to an increase in scale information. Similarly, the MSEs for both OBID and CFA decrease as the number of response categories increase; however, the MSE graphs for the five- and seven-point scales become very similar to each other across all sample sizes. It’s also expected that the MSEs for both approaches decrease as sample size increases, as a result of decreasing measurement errors. The asymptotic behavior of OBID is evaluated with sample size 500. As we expect, the two approaches produce almost identical MSEs with OBID being slightly smaller.

**Fig. 2:**
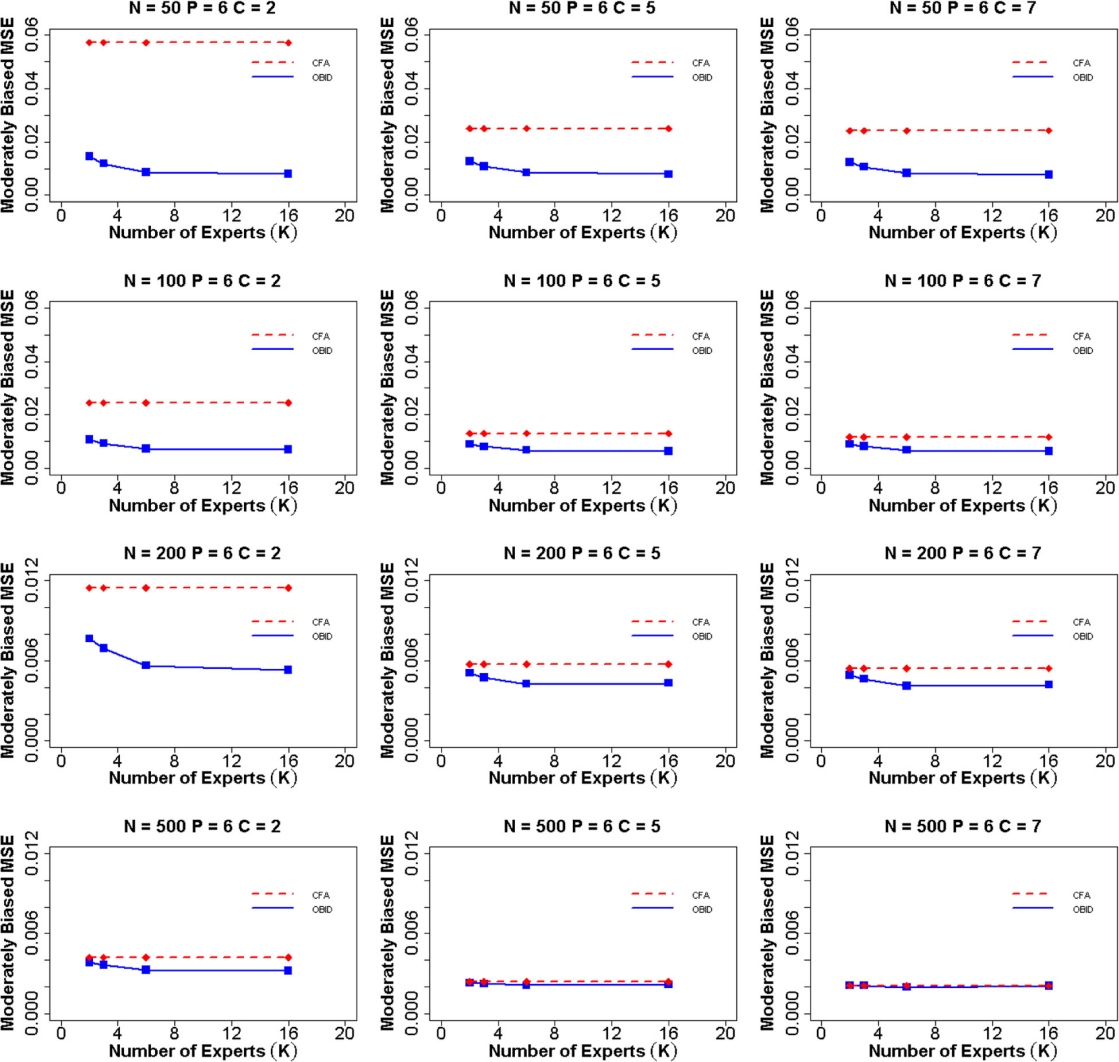
Average MSE of item-to-domain correlation $\rho$ for six items and moderately biased experts. Average mean squared error (MSE) for item-to-domain correlation $\rho$ using OBID (solid blue line) and ordinal CFA (dashed red line) when *P = 6* (number of items) and experts are moderately biased {${\rho}_0=\left(0.40,\;0.60,\;0.80,\;0.80,\;0.40,\;0.60\right)\Big\}$. The participant sample sizes are *N =* 50, 100, 200, and 500. The numbers of response categories are *C =* 2, 5, and 7, and the numbers of experts are *K =* 2, 3, 6, and 16. *Note*. OBID = Ordinal Bayesian Instrument Development; CFA = Confirmatory Factor Analysis

**Fig. 3:**
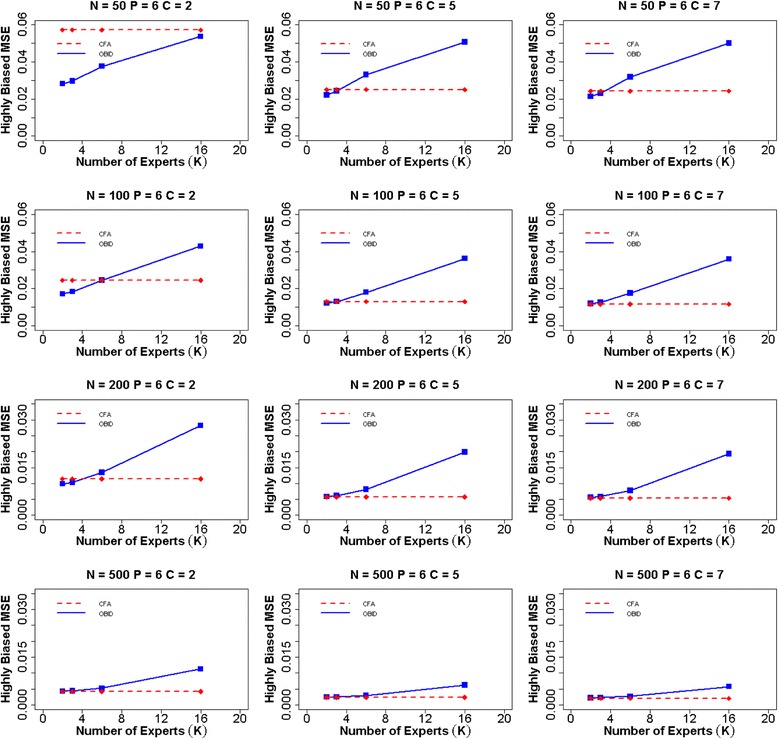
Average MSE of item-to-domain correlation $\rho$ for six items and highly biased experts. Average mean squared error (MSE) for item-to-domain correlation $\rho$ using OBID (solid blue line) and ordinal CFA (dashed red line) when *P = 6* (number of items) and experts are highly biased {${\rho}_0=\left(0.65,\;0.75,\;0.85,\;0.85,\;0.65,\;0.75\right)\Big\}$. The participant sample sizes are *N =* 50, 100, 200, and 500. The numbers of response categories are *C =* 2, 5, and 7, and the numbers of experts are *K =* 2, 3, 6, and 16. *Note*. OBID = Ordinal Bayesian Instrument Development; CFA = Confirmatory Factor Analysis

When experts are moderately biased (Fig. 2), a similar overall trend is observed as that of the unbiased case. OBID continues to outperform CFA in all scenarios; however, the differences in MSEs between OBID and CFA become smaller in the moderately biased case, indicating the effect of biased priors. Additionally, the efficiency gain of the OBID approach experiences a steady increase from 2–6 experts, and gradually levels off from 6–16 experts. This indicates that with moderately biased priors, having more than six experts does not contribute to any additional gain in the efficiency of OBID. When priors are highly biased (Fig. 3), our results support similar findings of BID [21] where the relative efficiency of OBID compared with CFA is a function of the number of experts. In the case of a binary response option and sample size 50, OBID produces smaller MSEs than CFA, despite of the receding efficiency as the number of experts increases. OBID is most efficient with smaller samples (e.g., *N* ≤ 100) and the number of experts is two or three. As number of experts increases, the impact of highly biased priors is substantial with smaller samples. The differences in MSEs between the OBID and CFA approaches exhibit similar patterns when the number of items is four or nine. MSE plots for additional simulation scenarios are included in Additional file 1: Figures S1–S6.

From simply observing the graphs, one may think that although OBID is more efficient, the performance of ordinal CFA is comparable and not a bad choice. However, a close examination of the frequency that ordinal CFA failed to converge and/or produced “bad” estimates (i.e., ${\rho}_j\notin \left[-1,\;1\right]$) reveals limitations of the classical method with small samples. In the six item simulation example, when *N* = 50 and *C =* 2, ordinal CFA fails to converge for 2 % of simulation iterations and produces out of bound correlation estimates for 21 % of simulation iterations. When both sample size and number of response categories increase, although all simulation iterations converge, CFA continues to produce 1-3 % out of bound correlation estimates. The four item scenarios face more challenges with convergence and reliable estimates with smaller samples. When the number of items is nine, the performance of CFA becomes more stable with only 6 % out of bound estimates in the sample size 50 and binary response option case. The complete table that summarizes CFA performance can be found in Additional file 1: Table S1. In contrast, the OBID approach consistently produces appropriate and reliable correlation estimates without any challenges using all sample sizes and response options.

**Fig. 4:**
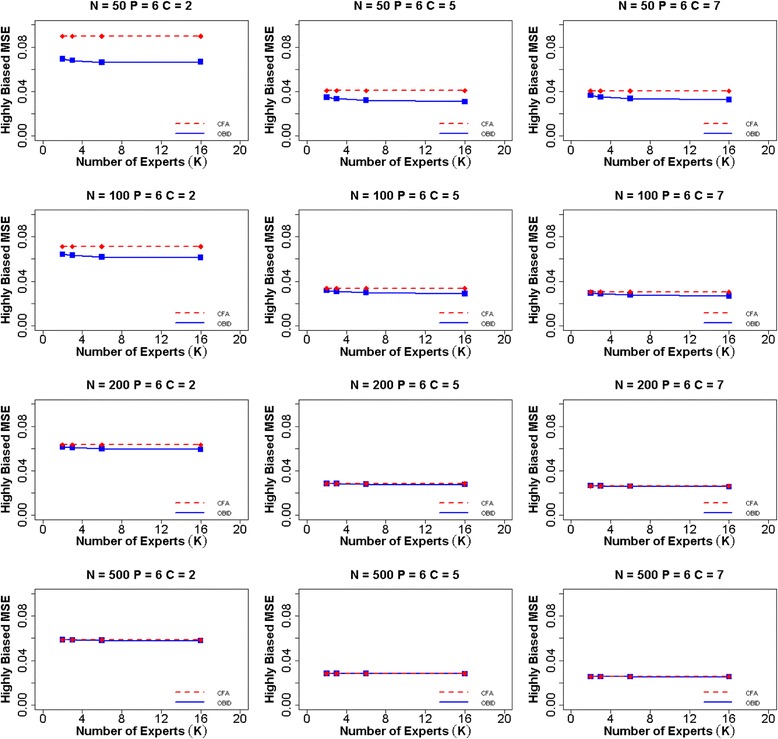
Average MSE of validity coefficient $\gamma$ for six items and highly biased experts. Mean squared error (MSE) for validity coefficient $\gamma$ using OBID (solid blue line) and ordinal CFA (dashed red line) when *P = 6* (number of items) and experts are highly biased {${\rho}_0=\left(0.65,\;0.75,\;0.85,\;0.85,\;0.65,\;0.75\right)\Big\}$. The participant sample sizes are *N =* 50, 100, 200, and 500. The numbers of response categories are *C =* 2, 5, and 7, and the numbers of experts are *K =* 2, 3, 6, and 16. *Note*. OBID = Ordinal Bayesian Instrument Development; CFA = Confirmatory Factor Analysis

Lastly we assessed the predictive validity of the two approaches under simulation settings. Under the previously mentioned assumption, the criterion is perfectly measured (i.e., the ideal target); thus the correlation of test scores ${f}_i$ (i.e., the ability parameter) and criterion scores is the same as the validity coefficient corrected for attenuation in the criterion only. Figure 4 displays the MSEs of the validity coefficient $\gamma$ computed using both OBID and CFA approaches when experts are highly biased and the number of items is six. Based on findings from Gajewski et al. [20], the subject experts tend to overestimate the relevancy of items, resulting in highly biased item-to-domain correlations. The predictive validity of OBID is examined in the extreme case of highly biased priors with a small sample size. For 50 participants, we can clearly observe that the MSE of OBID is the smallest with a binary response option (C = 2), compared with the CFA MSE. As number of response categories increases, OBID continues to have smaller MSE than that of CFA, although the differences become much smaller and almost negligible. When we increase the sample size, the two approaches become almost identical in terms of MSEs. A similar trend is observed in the four item and nine item scenarios, with corresponding plots included in Additional file 1: Figures S7–S14. Prior to the simulation, we hypothesize that $MSE\left({\gamma}_{OBID}\right)<MSE\left({\gamma}_{CFA}\right)$, ***f***_*OBID*_ is more correlated with ***f***^*T*^ than ***f***_*CFA*_. The simulation results support this original hypothesis. Thus, we make the conclusion that OBID produces higher predictive validity than that of the traditional approach, especially for small samples.

### Application to PAMS short form satisfaction survey data

Due to scarcely available mammography-specific satisfaction assessments, researchers at a Midwestern academic medical center developed the patient assessment of mammography services (PAMS) satisfaction survey (four-factor with 20 items) and PAMS-Short Form (single factor with seven items) [32]. In this section, we apply the OBID approach to complete data collected from the PAMS-Short Form instrument that was administered to 2865 women: Hispanic (36, 1.26 %), Non-Hispanic white (2768, 96.61 %), African American (34, 1.19 %), and other (27, 0.94 %). Participants rated their satisfaction with each of the seven items using a five-point Likert-type scale, ranging from “poor” to “excellent.” In addition, six subject experts were consulted and instructed to evaluate each of the seven items on a four-point relevancy scale. The University of Kansas Medical Center’s Internal Review Board (IRB) has determined that our study does not require oversight by the Human Subjects Committee (HSC), as data were collected for prior studies and they are provided to us in a de-identified fashion.

Based on the sample size for each racial/ethnic group, establishing construct validity evidence for scores for Non-Hispanic white participants is clearly adequate and traditional CFA will suffice based on the large sample. Yet, researchers are interested in establishing score-based construct validity evidence for groups such as Hispanic/African Americans which are typically small. Classical CFA is ill-suited for such small samples; thus we apply the OBID approach for the analyses of Hispanic/African American populations. For comparison purposes, we perform OBID with experts’ opinions (informative) and OBID without experts’ opinions (non-informative) due to estimation challenges with traditional CFA. Flat priors are assigned for the IRT model parameters in the OBID posterior non-informative cases, in which, ${\alpha}_j\sim N\left(0,\;1\right)$ and ${\lambda}_j\sim N\left(0,\;4\right)$. In addition, based on trial and error we set the tuning parameter value required for *MCMCpack* to 2.00 for both small populations. The estimated item-to-domain correlation ${\rho}_j$ and its corresponding standard error are reported in Additional file 1: Table S2.

The non-informative OBID tends to overestimate ${\rho}_j$ compared with the experts’ estimated correlations (.381-.673), for both Hispanic (.570-.920) and African American (.774-.942) populations. By integrating the experts’ opinions with participants’ data, informative OBID produces more reliable results (Hispanic: .466-.717; African American: .495-.725) by appropriately lowering the estimated ${\rho}_j$. Although not reported, the factor score or latent variable score for each participant (i.e., individual mammography satisfaction) also is estimated. Since the factor scores are adjusted or corrected for measurement error, patients can be more accurately classified into diagnostic groups based on factor scores, and then treated as covariates in subsequent analyses. The non-informative OBID estimates tend to have slightly smaller standard errors, which can be viewed as a trade-off between the overestimated reliability ${\rho}_j^2$ and the variance. Overall, as we expect, OBID successfully produces reliable item-to-domain correlation estimates and overcomes the small sample size challenge that often causes classical CFA to fail.

## Discussion

As health care moves rapidly toward a patient-centeredness care model, the development of reliable and valid PROMs is recognized as an essential step in promoting quality care. Despite of increasing public awareness, the development of PROMs using traditional psychometric methodologies often is lengthy and constrained by the large sample size requirement, resulting in substantially increased costs and resources. In this study, an innovative OBID approach within a Bayesian IRT framework is proposed to overcome both small sample size (e.g., patients from small populations or rare diseases) and ordinal data modeling limitations. OBID seamlessly and efficiently utilizes subject experts’ opinions (content validity) to form the prior distributions for the IRT parameters in construct validity analysis, as opposed to using arbitrarily selected priors in other Bayesian IRT simulation studies mentioned in the introduction.

A thorough comparison between OBID and traditional CFA is provided through assessing item-to-domain correlation estimates, MSEs, and predictive validity under a simulation setting with three different types of expert bias. Simulation results across all three types of expert bias clearly demonstrate that the overall performance of OBID is most superior to that of traditional CFA when the sample size is small (i.e., ≤ 100 participants) and the instrument response option is binary. When subject experts are biased, the gain in efficiency gradually recedes for OBID as number of experts increases; and traditional CFA eventually becomes more efficient. Although not discussed in the article, the average squared bias for the item-to-domain correlation estimate also is examined across different expert biases. The corresponding plots are included in Additional file 1: Figures S15–S23. A trade-off situation is observed as OBID may exhibit larger bias; yet it reduces the MSEs by decreasing variances. In addition, OBID produces higher predictive validity than that of the traditional method when the sample size is small. The simulation results are supported by the PAMS-Short Form example where OBID is successfully applied to small Hispanic and African American populations. The de-identified PAMS-Short Form data are available in a de-identified fashion to researchers upon request through e-mail to the corresponding author of this paper. Overall, while traditional methods are restricted by small samples, OBID proves to be an efficient and reliable approach.

One limitation of this study is associated with the source of experts’ information used in the PAMS-Short Form example. Opinions from the six content experts were originally consulted with the purpose of validating the PAMS instrument for the American Indian women population. Although the same set of survey items was administered to all American Indian, Hispanic, and African American populations, potential bias could be introduced due to the original focus of content experts. Nonetheless, as previously mentioned, reliable information collected from the six experts can still be utilized to form a “general prior” in establishing construct validity for Hispanic and African American populations. Another limitation of the study comes from the elicitation of content validity using relevance scales. Although Gajewski et al. [26] has demonstrated the appropriateness of measuring content validity using relevance scales, the equivalency with measuring content validity using correlation scales is approximate, which may have an effect on the parameter estimation. A third limitation of the study comes from the approximate normal distribution assumption that we made regarding the prior distribution of the experts after Fisher’s transformation. As pointed out by one of the reviewers, potential disagreements among selected subject experts may occur, which can cause the expert opinion to follow a bimodal (i.e., two groups of experts with opposite views) or even trimodal distribution. We acknowledge this limitation as this scenario was not examined in the current simulation study.

Two useful practical recommendations can be extracted from the current study. As previously mentioned, no standard method exist for determining appropriate tuning parameter values that ensure the 20-50 % acceptance rate needed for the MCMC procedure. Although trial and error also is used in this study, our findings provide a general guideline for the selection of tuning parameter values. We find that tuning parameter values 1.00, 0.70, 0.50, and 0.30 appear to work well for sample sizes 50, 100, 200, and 500, respectively. Additionally, our study results are consistent with findings from Polit and Beck [33] regarding the number of subject experts needed to establish content validity. Across three types of expert biases, results show that having more than six experts does not contribute to any additional gain in the efficiency of OBID. With highly biased experts, three experts appear to be sufficient for establishing content validity.

An implication from this study is that a hierarchical model can be considered in the future to incorporate the individual effect of content experts, as the scores experts assigned from item to item are likely to be correlated. In addition, the development of the user-friendly BID software can be used to guide the development of the OBID software, where multi-factor models can be evaluated, as it is common in many “long-form” questionnaires to encompass several constructs of interest. It is our ultimate goal to extend the application capability of OBID and present it as an efficient and reliable method for researchers and clinicians in future PROMs development.

## Conclusions

In this study, the efficiency of OBID is evaluated by comparing its performance to classical instrument development performance using actual and simulation data. This study successfully demonstrated that the OBID approach is more efficient than the classical approach when the sample size is small. OBID promises an efficient and reliable method for researchers and clinicians in future PROMs development for small populations or rare diseases.

## Additional file

Additional file 1:
**Additional Simulation and Application Results.** Additional simulation and application results referenced in Sections 3, 4 and 5. (PDF 901 kb)

## Abbreviations

AERA: American Educational Research Association
APA: American Psychological Association
BID: Bayesian Instrument Development
CFA: confirmatory factor analysis
FDA: Food and Drug Administration
HSC: Human Subjects Committee
IOM: Institute of Medicine
IACCV: Integrated Analysis of Content and Construct Validity
IRB: Internal Review Board
IRT: item response theory
MCMC: Markov chain Monte Carlo
MSE: Mean squared error
NCME: National Council on Measurement in Education
NIH: National Institute of Health
NQF: National Quality Forum
OBID: Ordinal Bayesian Instrument Development
PAMS: Patient assessment of mammography services
PCORI: Patient-Centered Outcomes Research Institute
PROMs: Patient-reported outcome measures
SEM: Structure equation modeling
DHHS: U. S. Department of Health and Human Services
WLSMV: Weighted least squares means- and variance-adjusted
