# Supplementary material for: A novel method for expediting the development of patient-reported outcome measures and an evaluation of its performance via simulation
Source: BMC Med Res Methodol. 2015 Sep 29;15:77. doi: 10.1186/s12874-015-0071-5 (PMC4589027; doi:10.1186/s12874-015-0071-5)
Supplement: Additional file 1: — Additional Simulation and Application Results. Additional simulation and application results referenced in Sections 3, 4 and 5. (PDF 901 kb) [file 12874_2015_71_MOESM1_ESM.pdf]

Additional file for "A Novel Method for Expediting the Development of Patient Reported Outcome Measures and an Evaluation of Its Performance via Simulation" by Lili Garrard, Larry R. Price, Marjorie J. Bott, and Byron J. Gajewski

Table 1. Percent of CFA simulation iterations that fail to converge and/or produce out of bound item-to-domain correlation (i.e., $\rho_j \notin [-1, 1]$).

Number of Items (P)	Number of Participants (N)	Number of Response Categories (C)	CFA Fail to Converge (%)	CFA Out of Bound Estimate (%)
4	50	2	6	21
	50	5	1	13
	50	7	0	14
	100	2	3	14
	100	5	0	3
	100	7	1	4
	200	2	2	5
	200	5	0	1
	200	7	0	1
	500	2	0	1
	500	5	0	0
	500	7	0	0
6	50	2	2	21
	50	5	0	2
	50	7	0	2
	100	2	0	3
	100	5	0	0
	100	7	0	1
	200	2	0	2
	200	5	0	0
	200	7	0	0
	500	2	0	0
	500	5	0	0
	500	7	0	0
9	50	2	0	6
	50	5	0	0
	50	7	0	0
	100	2	0	0
	100	5	0	0
	100	7	0	0
	200	2	0	0
	200	5	0	0
	200	7	0	0
	500	2	0	0
	500	5	0	0
	500	7	0	0

Table 2. Item-to-domain correlation ρ estimates and standard errors for prior (content experts), OBID posterior informative (experts information used), and OBID posterior non-informative (experts information not used).

Item	Expert Prior	Hispanic (*N*=36)		African American (*N*=34)	
		OBID (Posterior Informative)	OBID (Posterior Non-informative)	OBID (Posterior Informative)	OBID (Posterior Non-informative)
Item 1	0.381 (0.130)	0.466 (0.093)	0.710 (0.123)	0.495 (0.086)	0.774 (0.102)
Item 2	0.673 (0.112)	0.565 (0.118)	0.570 (0.160)	0.674 (0.088)	0.791 (0.094)
Item 3	0.472 (0.119)	0.615 (0.074)	0.914 (0.055)	0.653 (0.066)	0.942 (0.036)
Item 4	0.629 (0.109)	0.717 (0.070)	0.920 (0.053)	0.718 (0.068)	0.884 (0.059)
Item 5	0.528 (0.116)	0.537 (0.097)	0.607 (0.159)	0.641 (0.074)	0.908 (0.056)
Item 6	0.562 (0.110)	0.647 (0.079)	0.783 (0.110)	0.620 (0.077)	0.819 (0.079)
Item 7	0.561 (0.118)	0.653 (0.082)	0.784 (0.110)	0.725 (0.062)	0.938 (0.037)

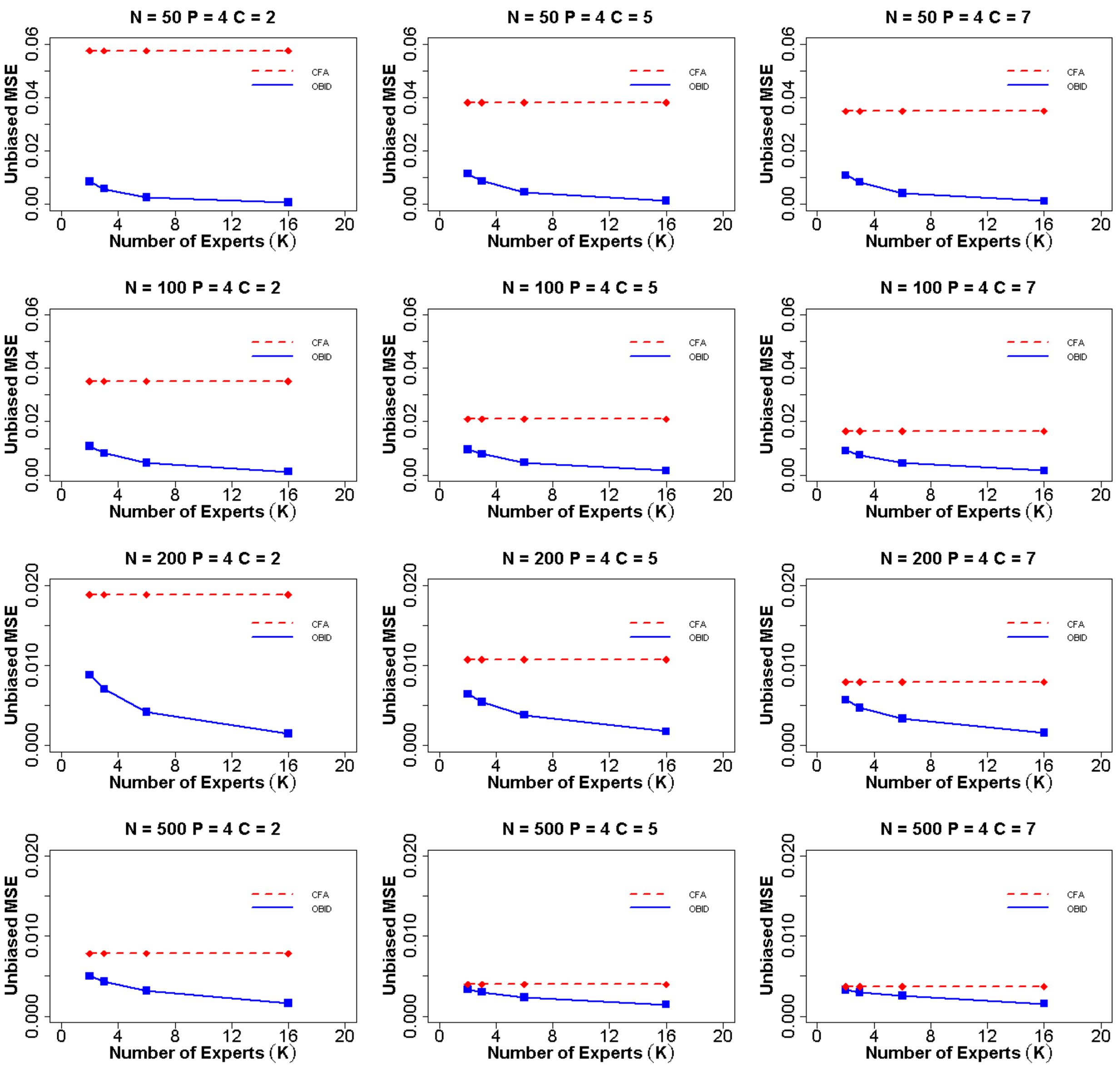

Figure 1. Average mean squared error (MSE) for item-to-domain correlation ρ using OBID (solid blue line) and ordinal CFA (dashed red line) when $P = 4$ (number of items) and experts are unbiased $\{\rho_0 = (0.50, 0.30, 0.70, 0.50)\}$. The participant sample sizes are N = 50, 100, 200, and 500. The numbers of response categories are C = 2, 5, and 7, and the numbers of experts are K = 2, 3, 6, and 16.

Note. OBID = Ordinal Bayesian Instrument Development; CFA = Confirmatory Factor Analysis.

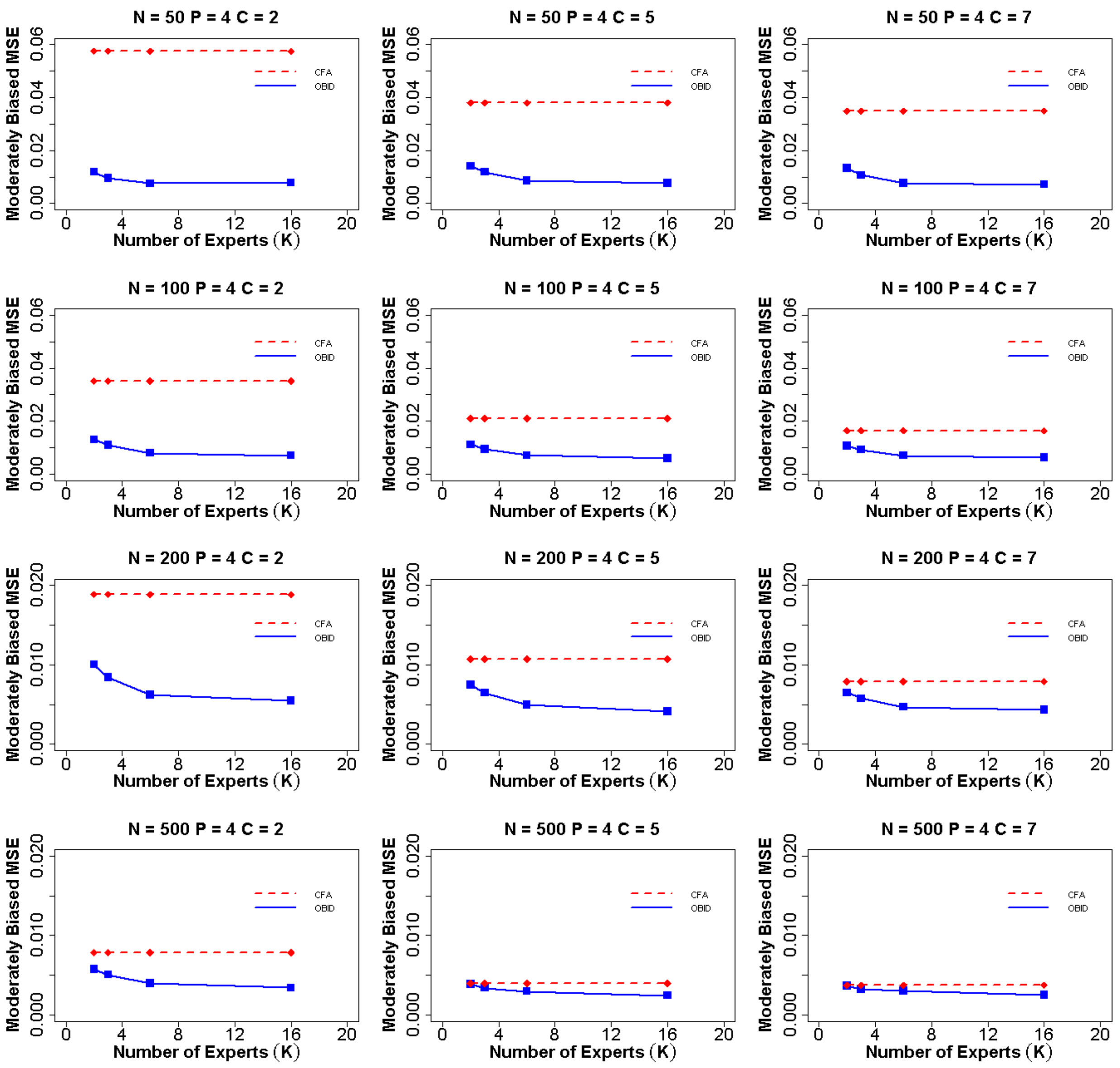

Figure 2. Average mean squared error (MSE) for item-to-domain correlation ρ using OBID (solid blue line) and ordinal CFA (dashed red line) when *P* = *4* (number of items) and experts are moderately biased $\{\rho_0 = (0.60, 0.40, 0.80, 0.60)\}$. The participant sample sizes are *N* = 50, 100, 200, and 500. The numbers of response categories are *C* = 2, 5, and 7, and the numbers of experts are *K* = 2, 3, 6, and 16.

Note. OBID = Ordinal Bayesian Instrument Development; CFA = Confirmatory Factor Analysis.

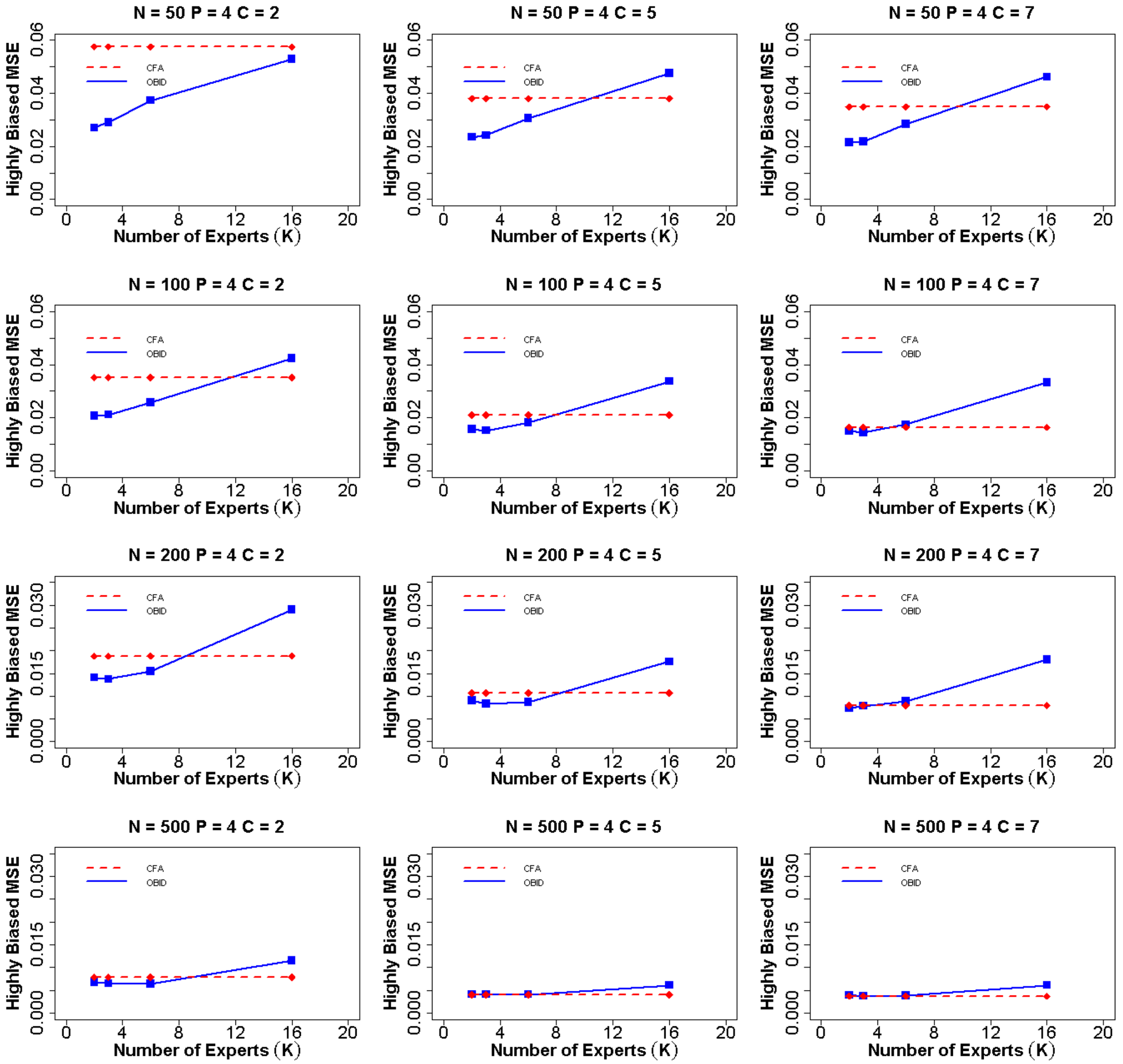

Figure 3. Average mean squared error (MSE) for item-to-domain correlation ρ using OBID (solid blue line) and ordinal CFA (dashed red line) when $P = 4$ (number of items) and experts are highly biased $\{\rho_0 = (0.75, 0.65, 0.85, 0.75)\}$. The participant sample sizes are N = 50, 100, 200, and 500. The numbers of response categories are C = 2, 5, and 7, and the numbers of experts are K = 2, 3, 6, and 16.

Note. OBID = Ordinal Bayesian Instrument Development; CFA = Confirmatory Factor Analysis.

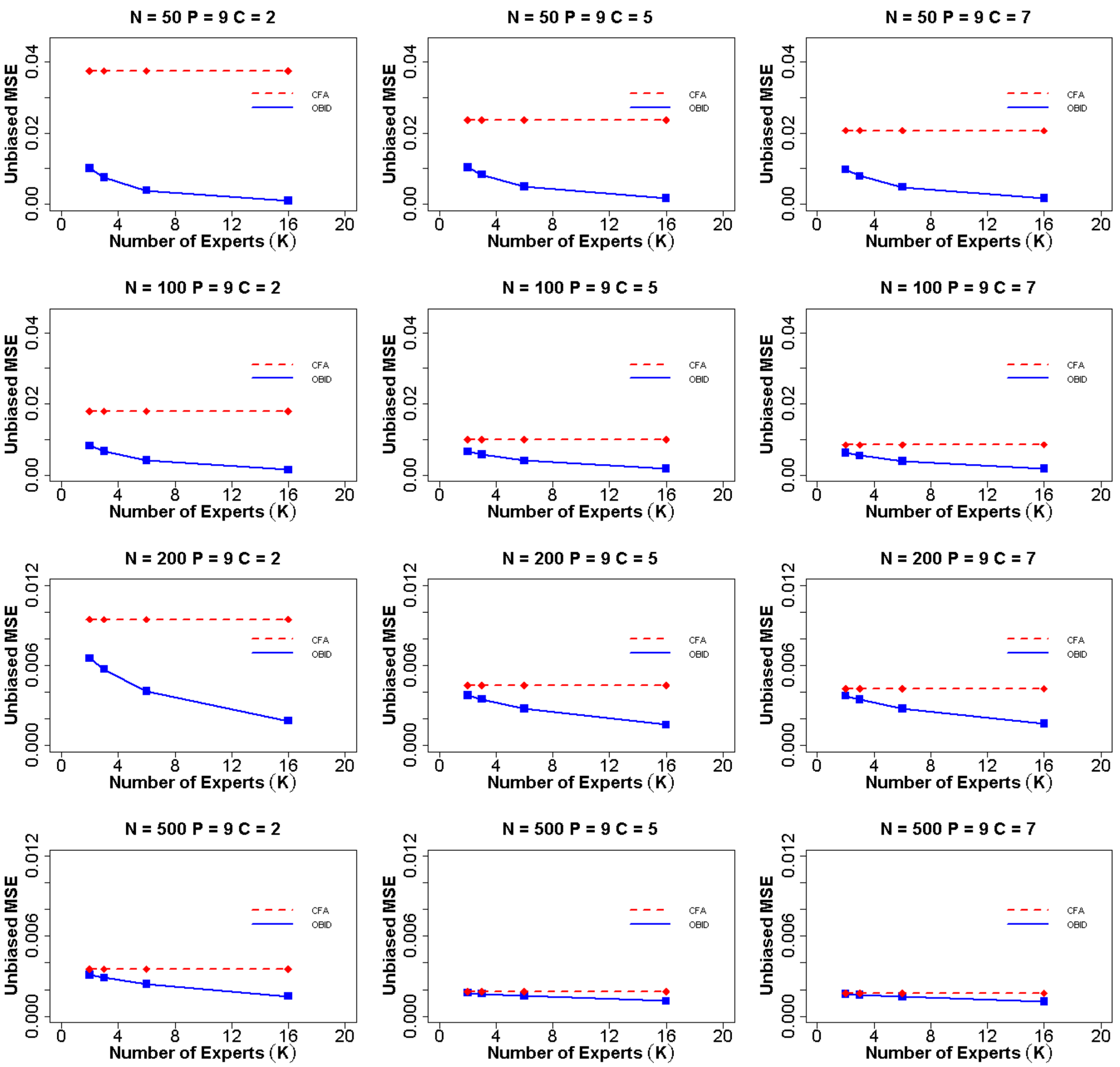

Figure 4. Average mean squared error (MSE) for item-to-domain correlation ρ using OBID (solid blue line) and ordinal CFA (dashed red line) when $P = 9$ (number of items) and experts are unbiased $\{\rho_0 = (0.30, 0.50, 0.70, 0.70, 0.30, 0.50, 0.70, 0.50, 0.30)\}$. The participant sample sizes are N = 50, 100, 200, and 500. The numbers of response categories are C = 2, 5, and 7, and the numbers of experts are K = 2, 3, 6, and 16.

Note. OBID = Ordinal Bayesian Instrument Development; CFA = Confirmatory Factor Analysis.

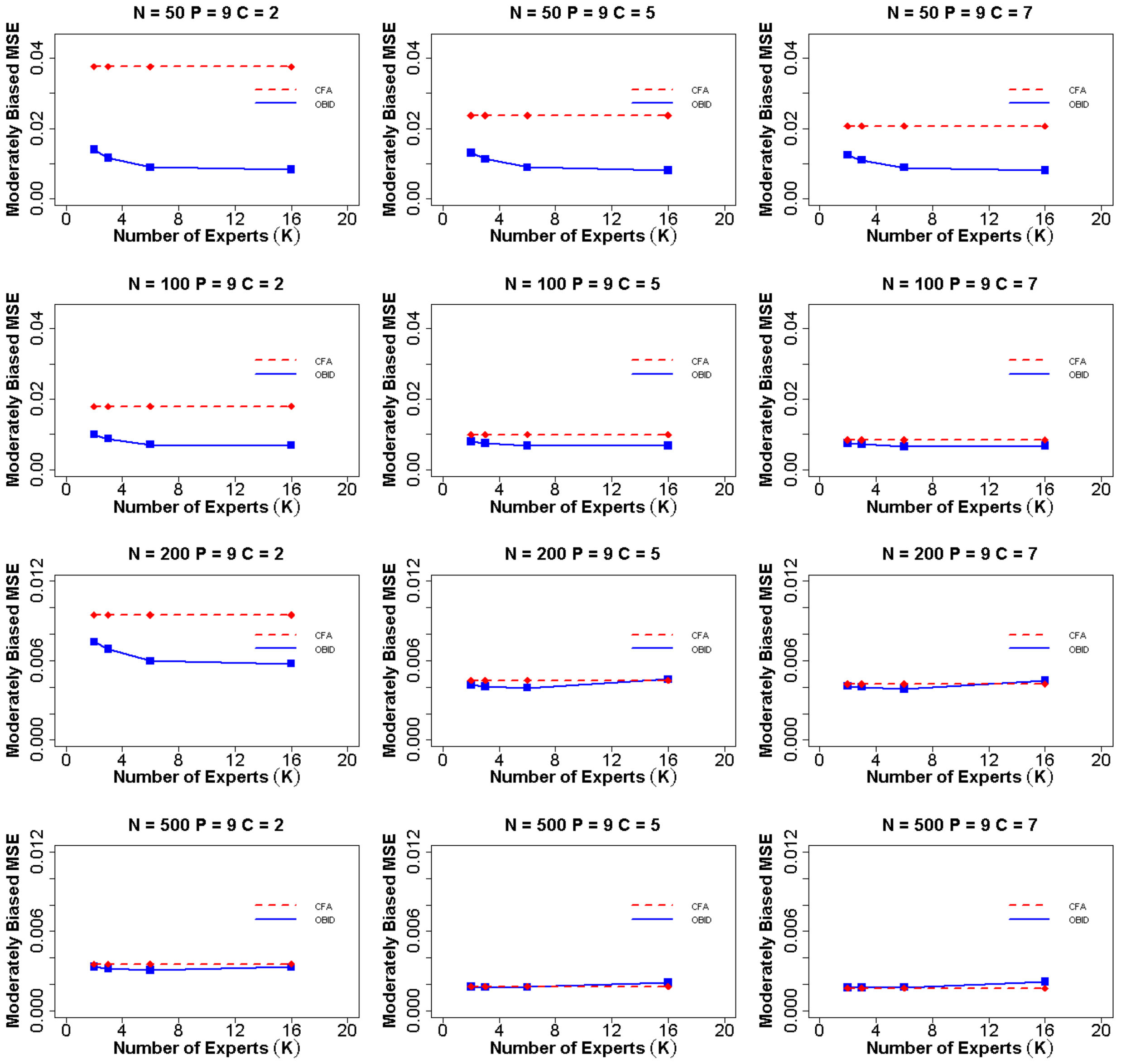

Figure 5. Average mean squared error (MSE) for item-to-domain correlation ρ using OBID (solid blue line) and ordinal CFA (dashed red line) when $P = 9$ (number of items) and experts are moderately biased $\{\rho_0 = (0.40, 0.60, 0.80, 0.80, 0.40, 0.60, 0.80, 0.60, 0.40)\}$. The participant sample sizes are N = 50, 100, 200, and 500. The numbers of response categories are C = 2, 5, and 7, and the numbers of experts are K = 2, 3, 6, and 16.

Note. OBID = Ordinal Bayesian Instrument Development; CFA = Confirmatory Factor Analysis.

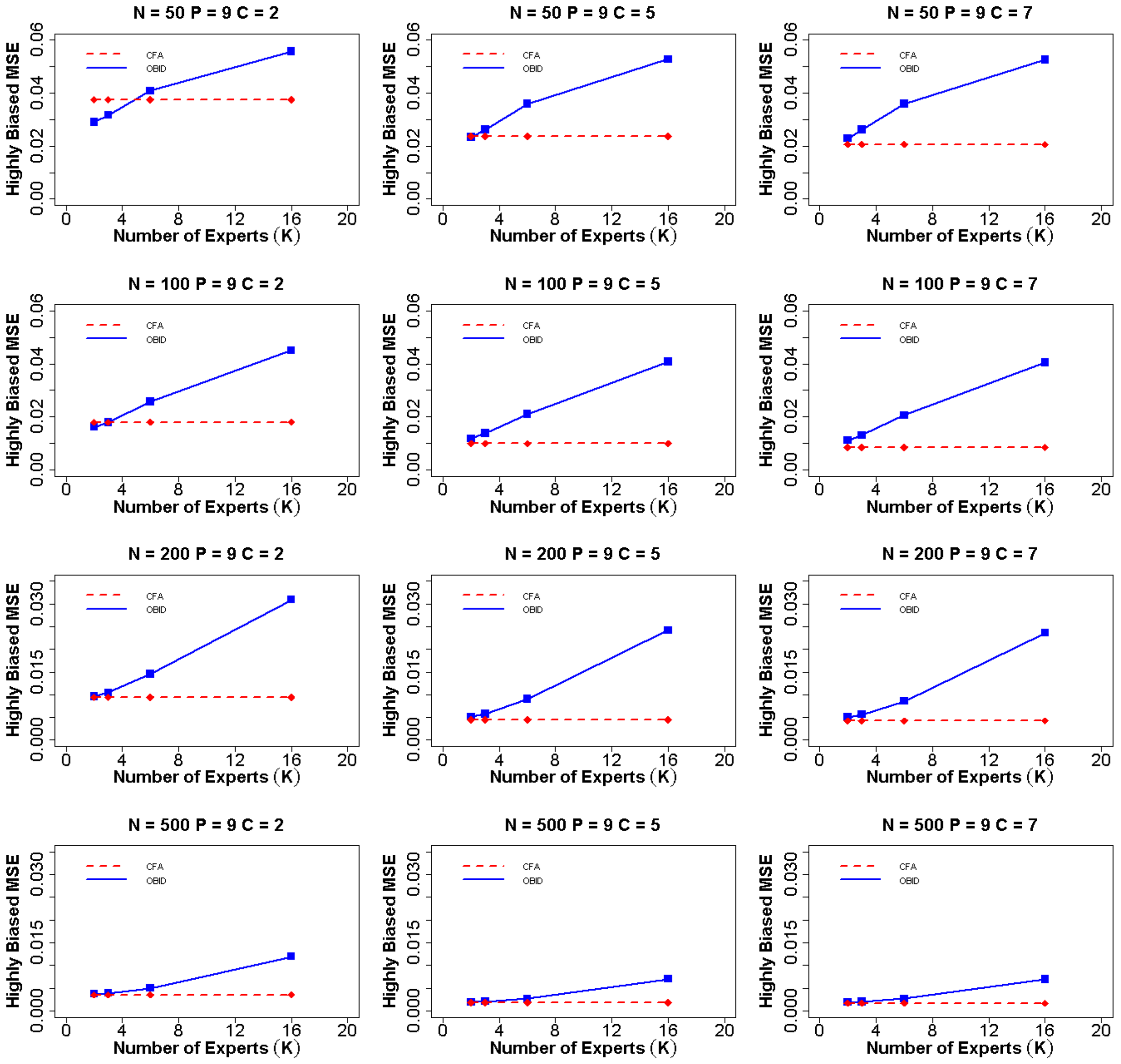

Figure 6. Average mean squared error (MSE) for item-to-domain correlation ρ using OBID (solid blue line) and ordinal CFA (dashed red line) when $P = 9$ (number of items) and experts are highly biased $\{\rho_0 = (0.65,0.75,0.85,0.85,0.65,0.75,0.85,0.75,0.65)\}$. The participant sample sizes are N = 50, 100, 200, and 500. The numbers of response categories are C = 2, 5, and 7, and the numbers of experts are K = 2, 3, 6, and 16.

Note. OBID = Ordinal Bayesian Instrument Development; CFA = Confirmatory Factor Analysis.

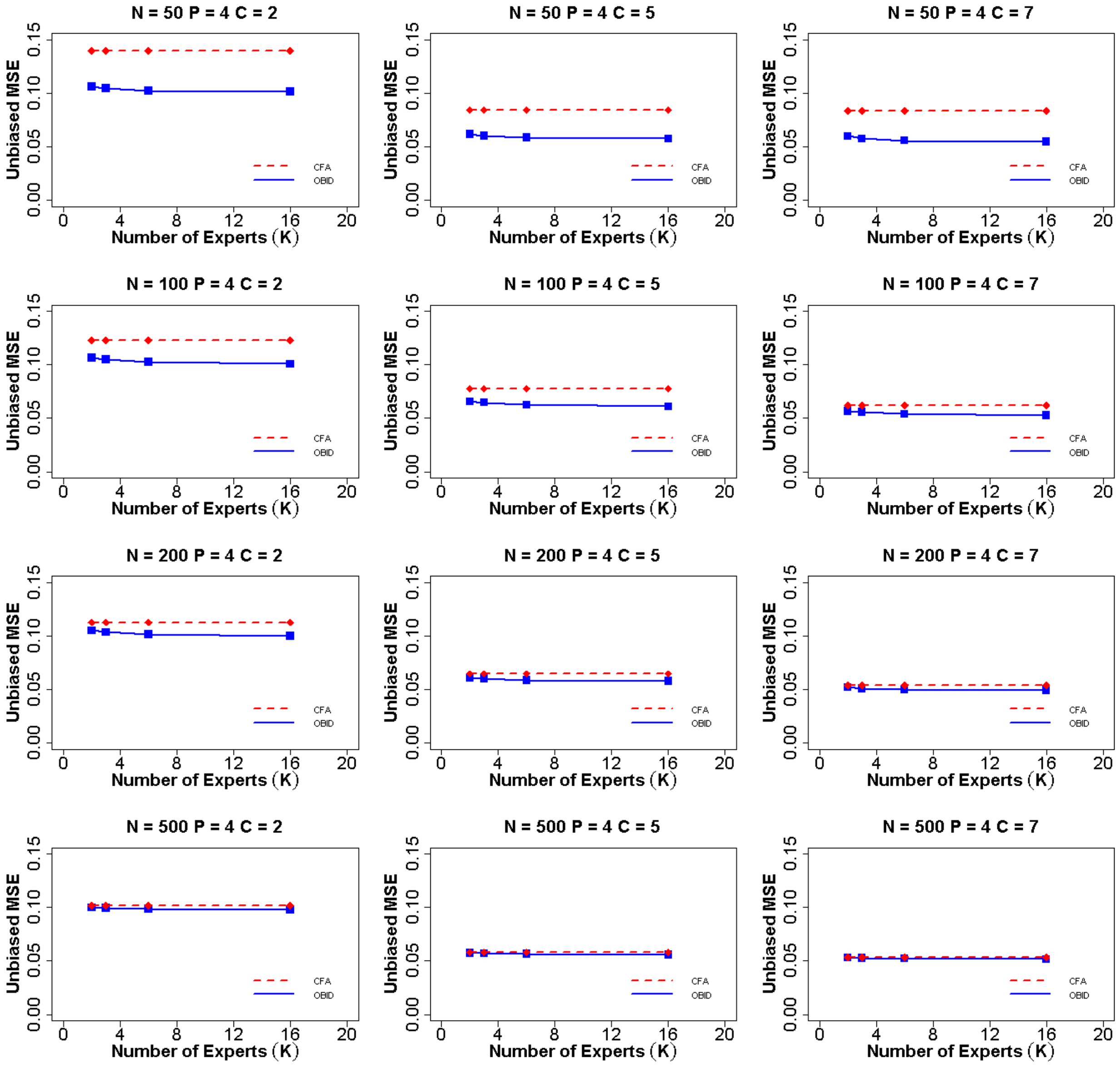

Figure 7. Mean squared error (MSE) for validity coefficient γ using OBID (solid blue line) and ordinal CFA (dashed red line) when $P = 4$ (number of items) and experts are unbiased $\{\rho_0 = (0.50, 0.30, 0.70, 0.50)\}$. The participant sample sizes are N = 50, 100, 200, and 500. The numbers of response categories are C = 2, 5, and 7, and the numbers of experts are K = 2, 3, 6, and 16.

Note. OBID = Ordinal Bayesian Instrument Development; CFA = Confirmatory Factor Analysis.

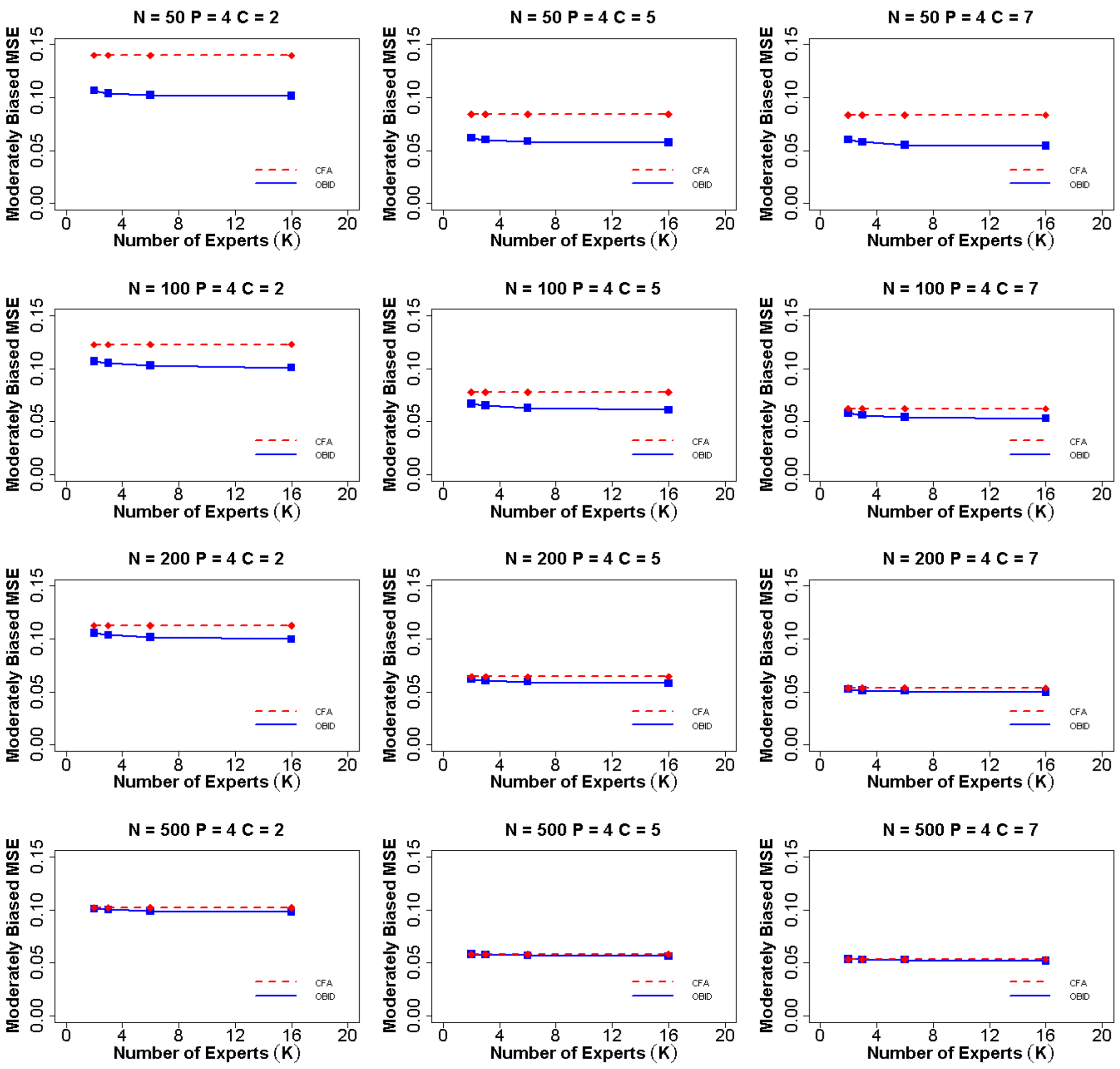

Figure 8. Average mean squared error (MSE) for validity coefficient γ using OBID (solid blue line) and ordinal CFA (dashed red line) when $P = 4$ (number of items) and experts are moderately biased $\{\rho_0 = (0.60, 0.40, 0.80, 0.60)\}$. The participant sample sizes are N = 50, 100, 200, and 500. The numbers of response categories are C = 2, 5, and 7, and the numbers of experts are K = 2, 3, 6, and 16.

Note. OBID = Ordinal Bayesian Instrument Development; CFA = Confirmatory Factor Analysis.

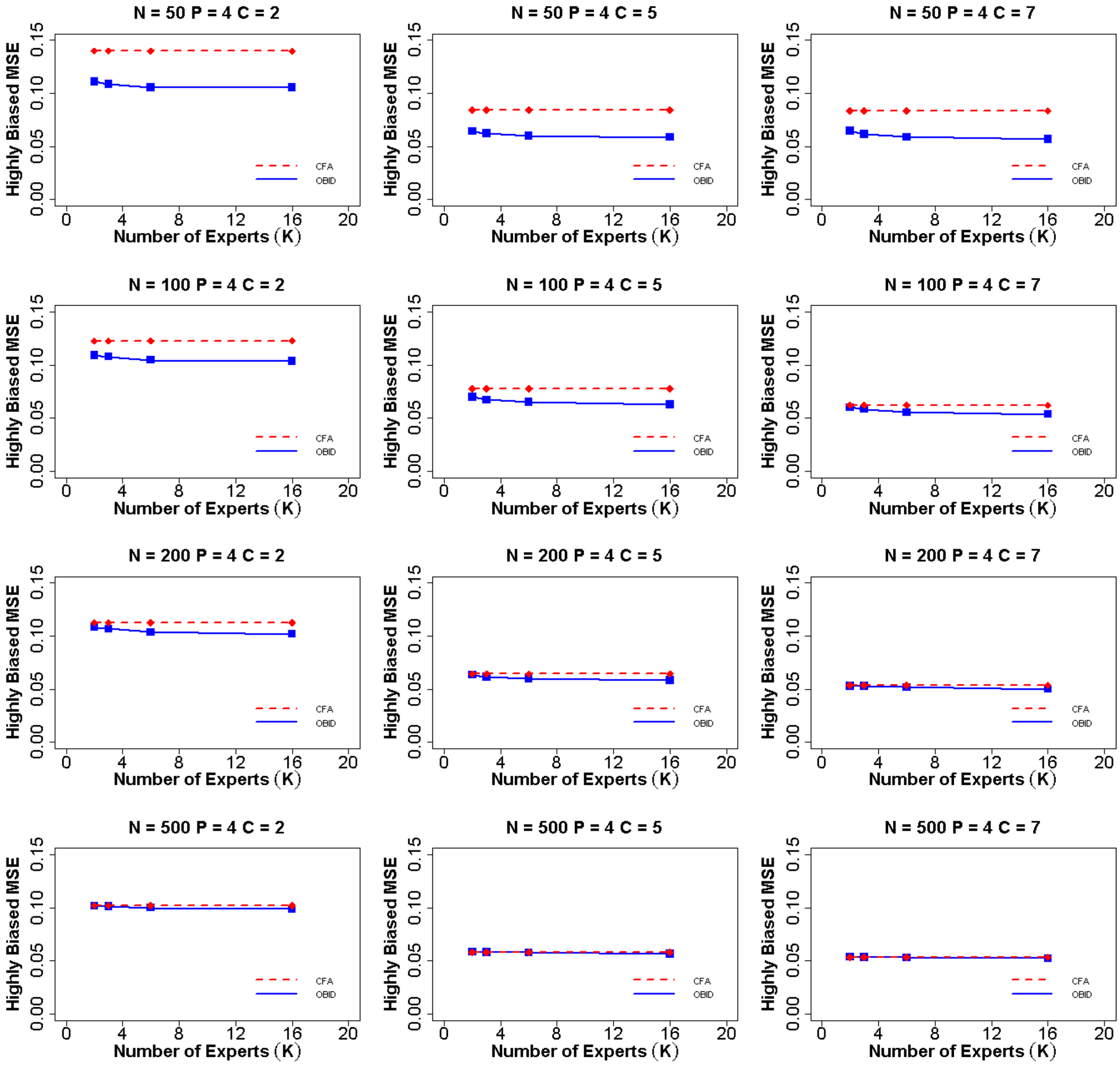

Figure 9. Average mean squared error (MSE) for validity coefficient γ using OBID (solid blue line) and ordinal CFA (dashed red line) when $P = 4$ (number of items) and experts are highly biased $\{\rho_0 = (0.75, 0.65, 0.85, 0.75)\}$. The participant sample sizes are N = 50, 100, 200, and 500. The numbers of response categories are C = 2, 5, and 7, and the numbers of experts are K = 2, 3, 6, and 16.

Note. OBID = Ordinal Bayesian Instrument Development; CFA = Confirmatory Factor Analysis.

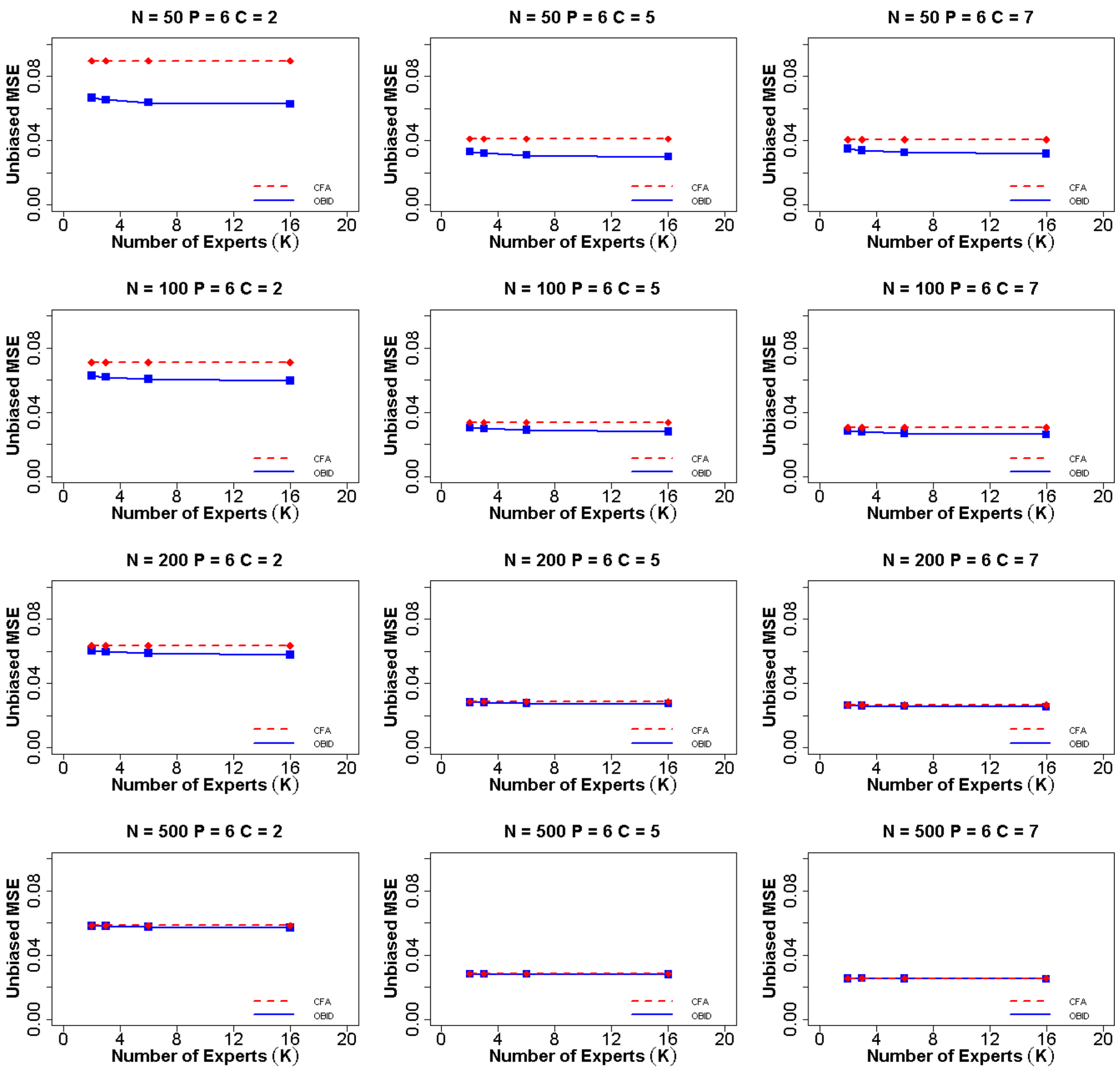

Figure 10. Average mean squared error (MSE) for validity coefficient γ using OBID (solid blue line) and ordinal CFA (dashed red line) when P = *6* (number of items) and experts are unbiased $\{\rho_0 = (0.30, 0.50, 0.70, 0.70, 0.30, 0.50)\}$. The participant sample sizes are N = 50, 100, 200, and 500. The numbers of response categories are C = 2, 5, and 7, and the numbers of experts are K = 2, 3, 6, and 16.

Note. OBID = Ordinal Bayesian Instrument Development; CFA = Confirmatory Factor Analysis.

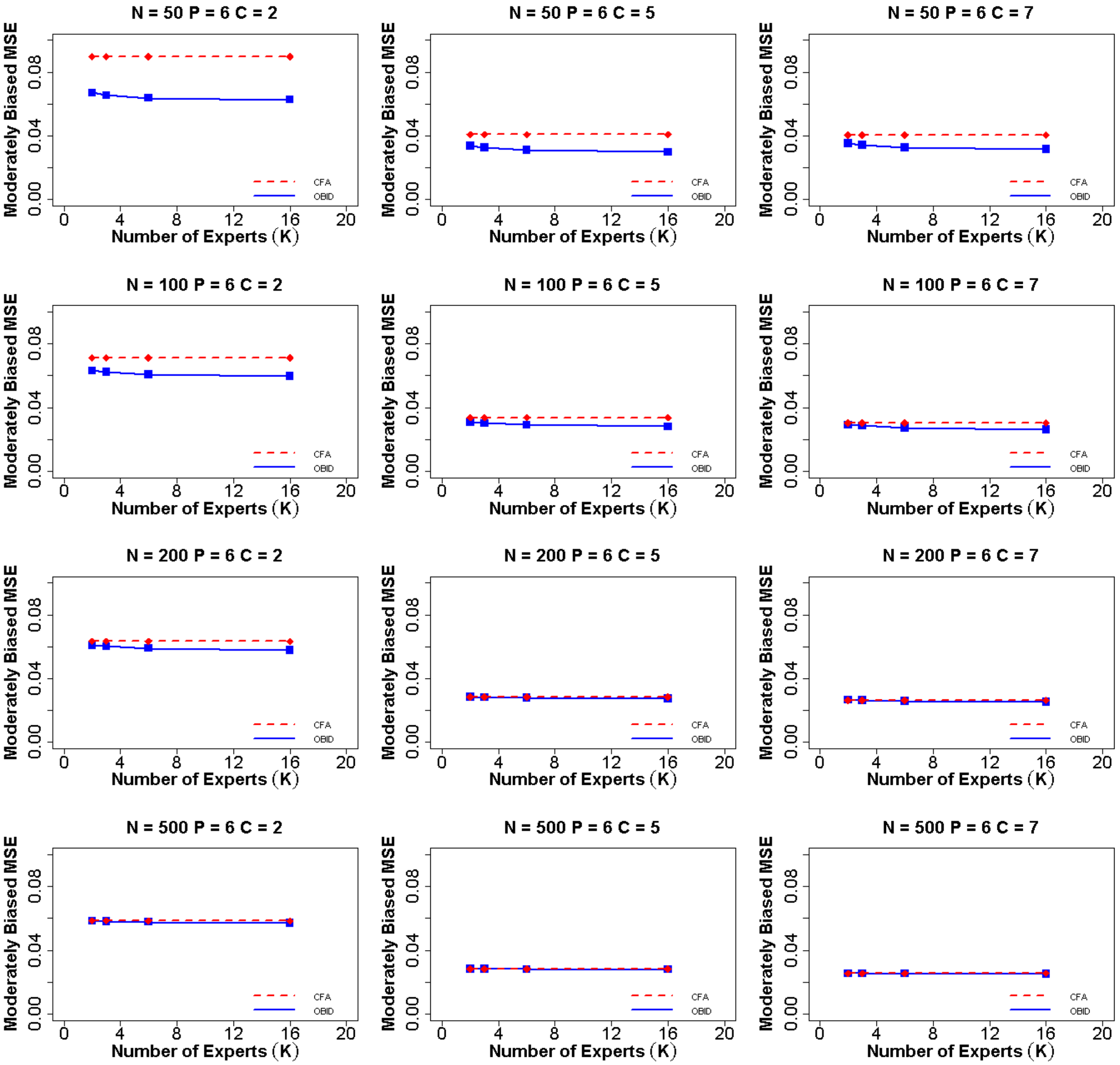

Figure 11. Average mean squared error (MSE) for validity coefficient γ using OBID (solid blue line) and ordinal CFA (dashed red line) when $P = 6$ (number of items) and experts are moderately biased $\{\rho_0 = (0.40, 0.60, 0.80, 0.80, 0.40, 0.60)\}$. The participant sample sizes are N = 50, 100, 200, and 500. The numbers of response categories are C = 2, 5, and 7, and the numbers of experts are K = 2, 3, 6, and 16.

Note. OBID = Ordinal Bayesian Instrument Development; CFA = Confirmatory Factor Analysis.

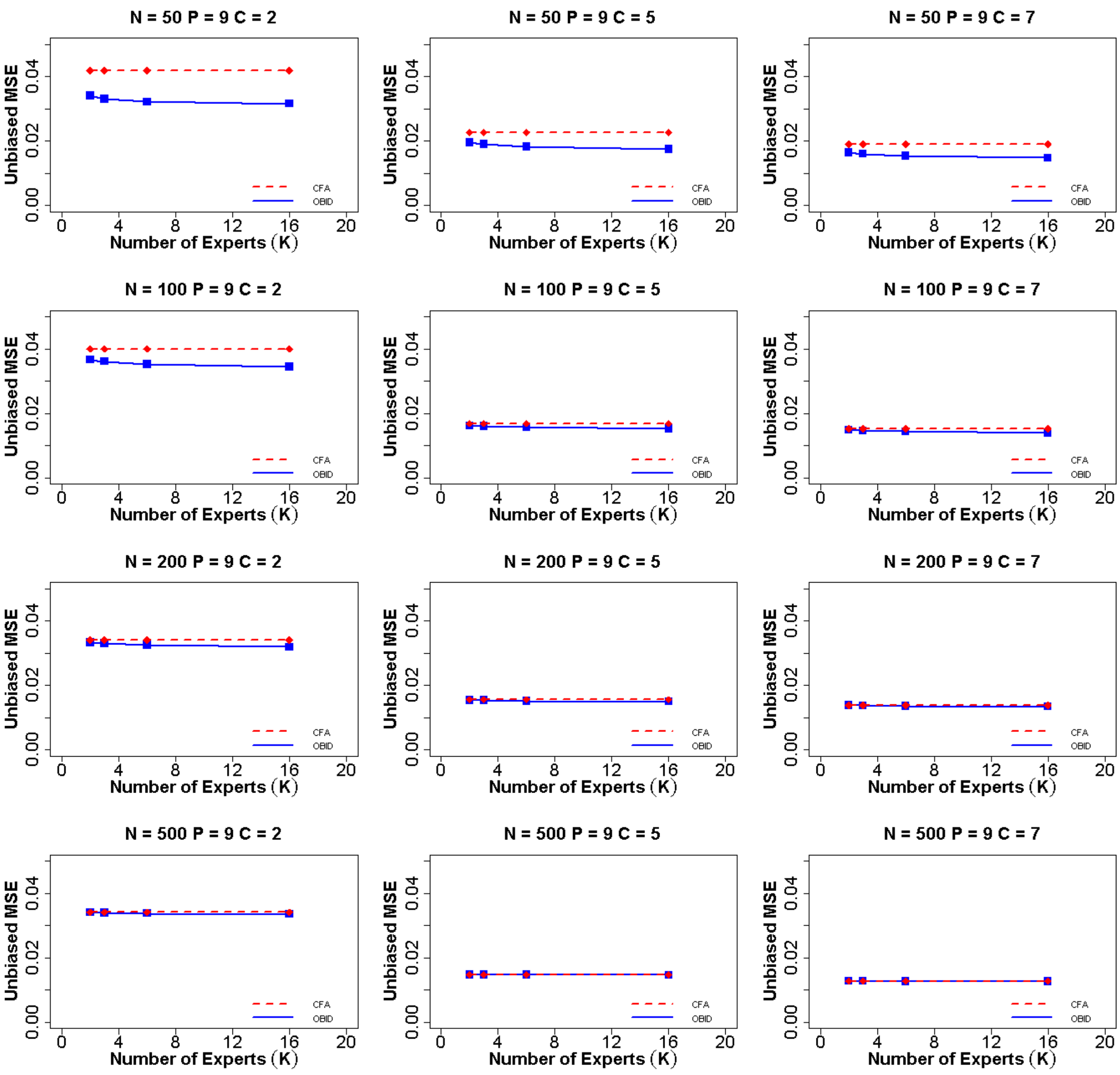

Figure 12. Average mean squared error (MSE) for validity coefficient γ using OBID (solid blue line) and ordinal CFA (dashed red line) when $P = 9$ (number of items) and experts are unbiased $\{\rho_0 = (0.30, 0.50, 0.70, 0.70, 0.30, 0.50, 0.70, 0.50, 0.30)\}$. The participant sample sizes are N = 50, 100, 200, and 500. The numbers of response categories are C = 2, 5, and 7, and the numbers of experts are K = 2, 3, 6, and 16.

Note. OBID = Ordinal Bayesian Instrument Development; CFA = Confirmatory Factor Analysis.

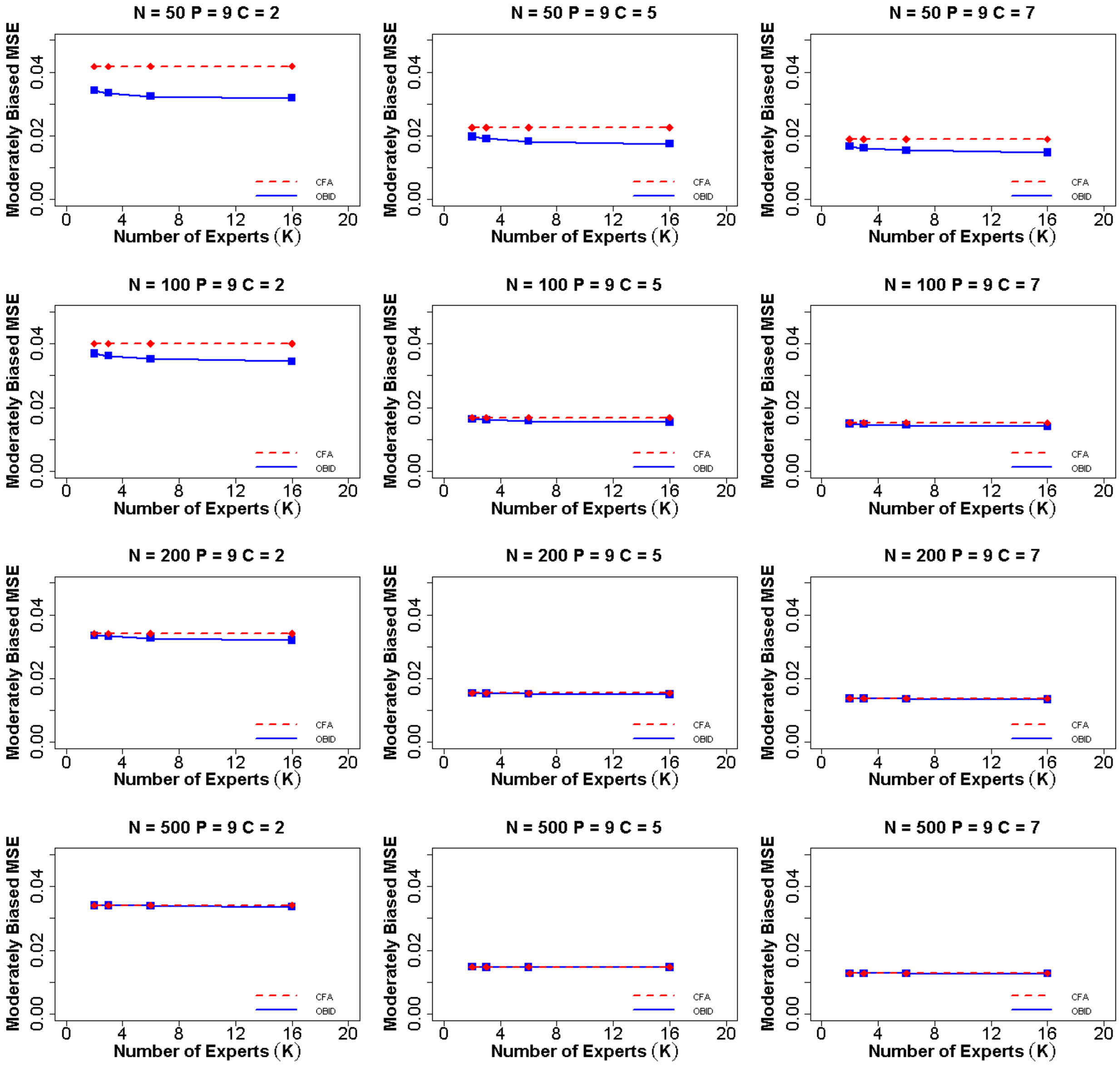

Figure 13. Average mean squared error (MSE) for validity coefficient γ using OBID (solid blue line) and ordinal CFA (dashed red line) when $P = 9$ (number of items) and experts are moderately biased $\{\rho_0 = (0.40, 0.60, 0.80, 0.80, 0.40, 0.60, 0.80, 0.60, 0.40)\}$. The participant sample sizes are N = 50, 100, 200, and 500. The numbers of response categories are C = 2, 5, and 7, and the numbers of experts are K = 2, 3, 6, and 16.

Note. OBID = Ordinal Bayesian Instrument Development; CFA = Confirmatory Factor Analysis.

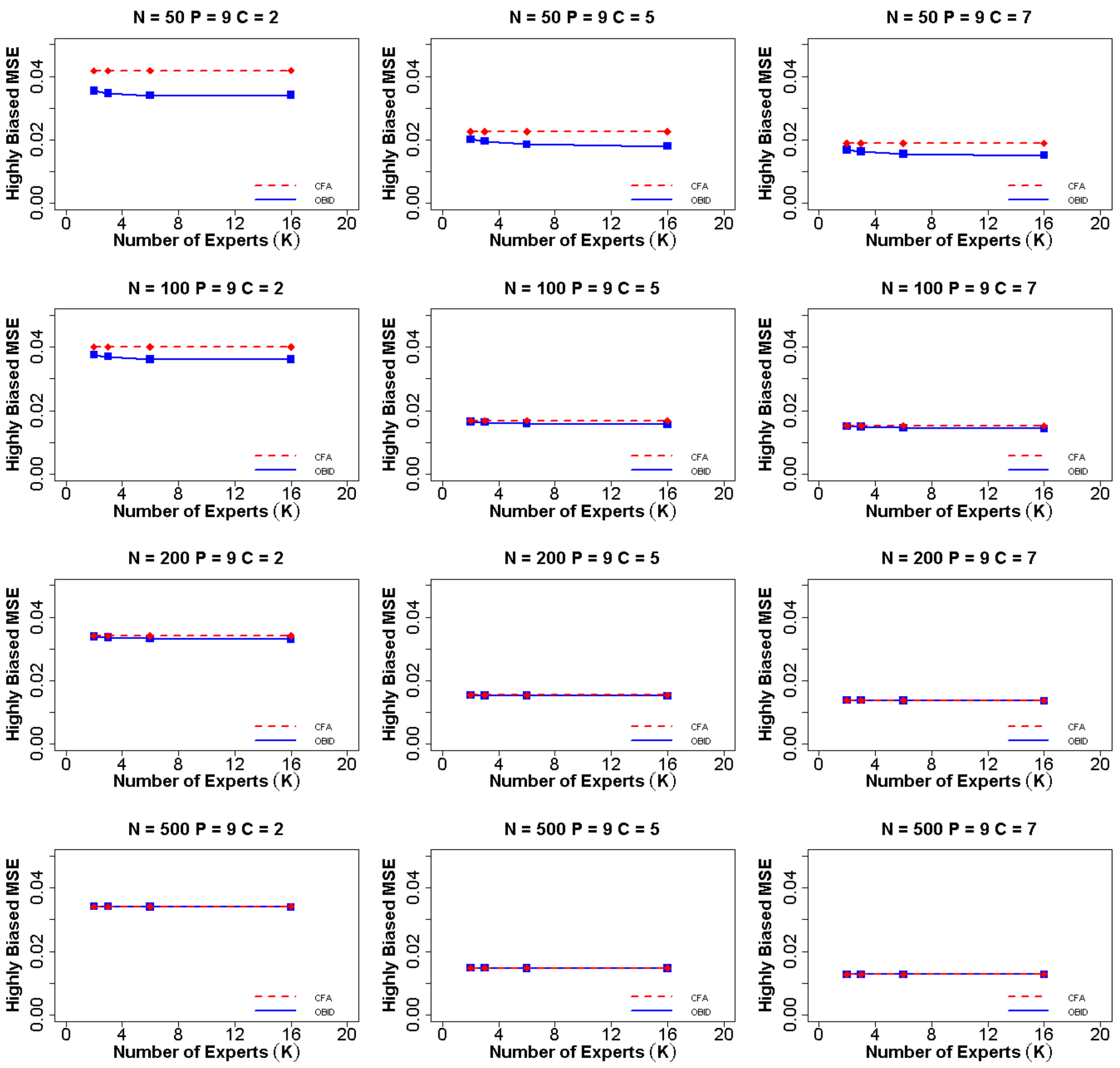

Figure 14. Average mean squared error (MSE) for validity coefficient γ using OBID (solid blue line) and ordinal CFA (dashed red line) when $P = 9$ (number of items) and experts are highly biased $\{\rho_0 = (0.65,0.75,0.85,0.85,0.65,0.75,0.85,0.75,0.65)\}$. The participant sample sizes are N = 50, 100, 200, and 500. The numbers of response categories are C = 2, 5, and 7, and the numbers of experts are K = 2, 3, 6, and 16.

Note. OBID = Ordinal Bayesian Instrument Development; CFA = Confirmatory Factor Analysis.

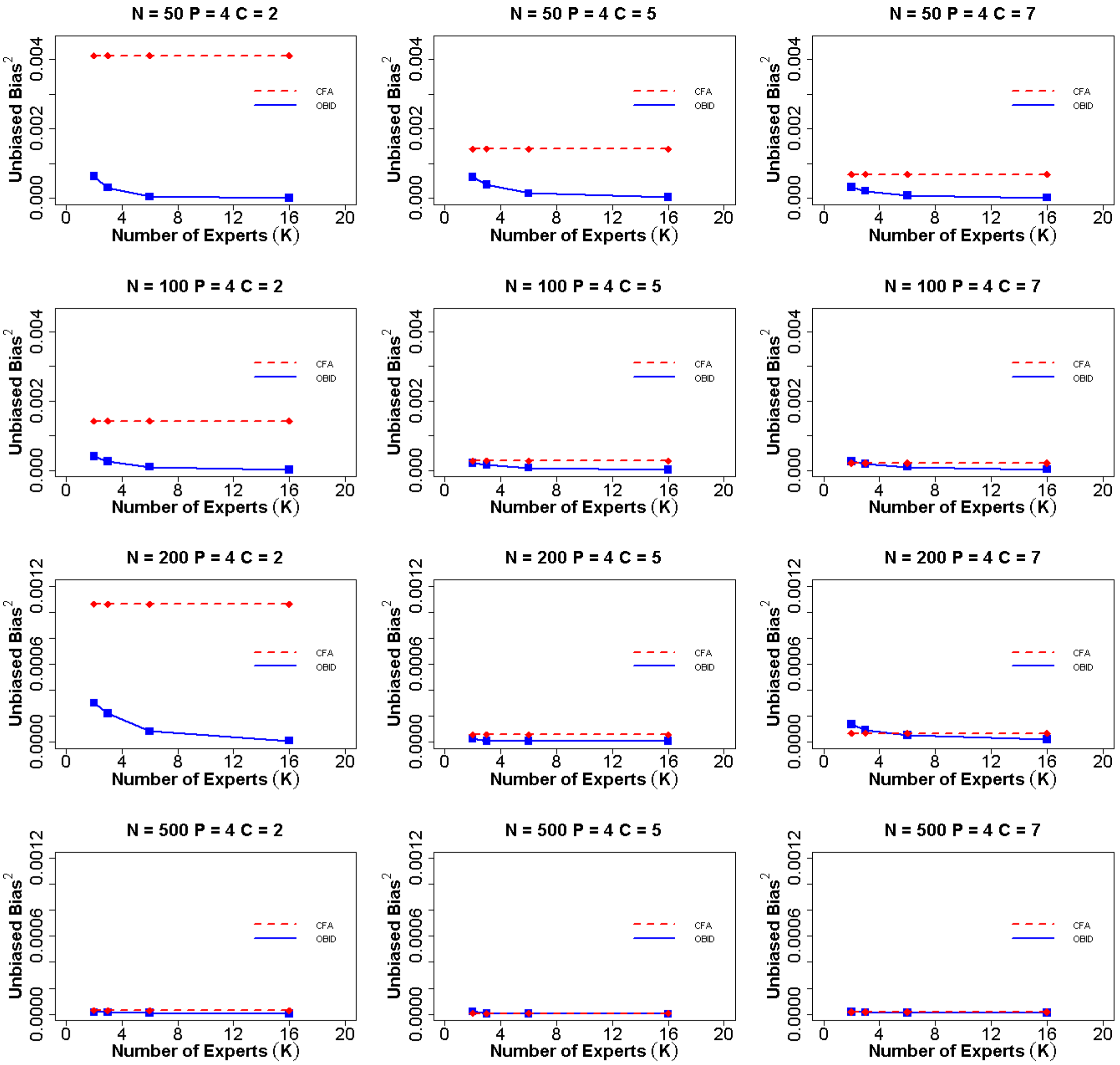

Figure 15. Average squared bias for item-to-domain correlation ρ using OBID (solid blue line) and ordinal CFA (dashed red line) when $P = 4$ (number of items) and experts are unbiased $\{\rho_0 = (0.50, 0.30, 0.70, 0.50)\}$. The participant sample sizes are N = 50, 100, 200, and 500. The numbers of response categories are C = 2, 5, and 7, and the numbers of experts are K = 2, 3, 6, and 16.

Note. OBID = Ordinal Bayesian Instrument Development; CFA = Confirmatory Factor Analysis.

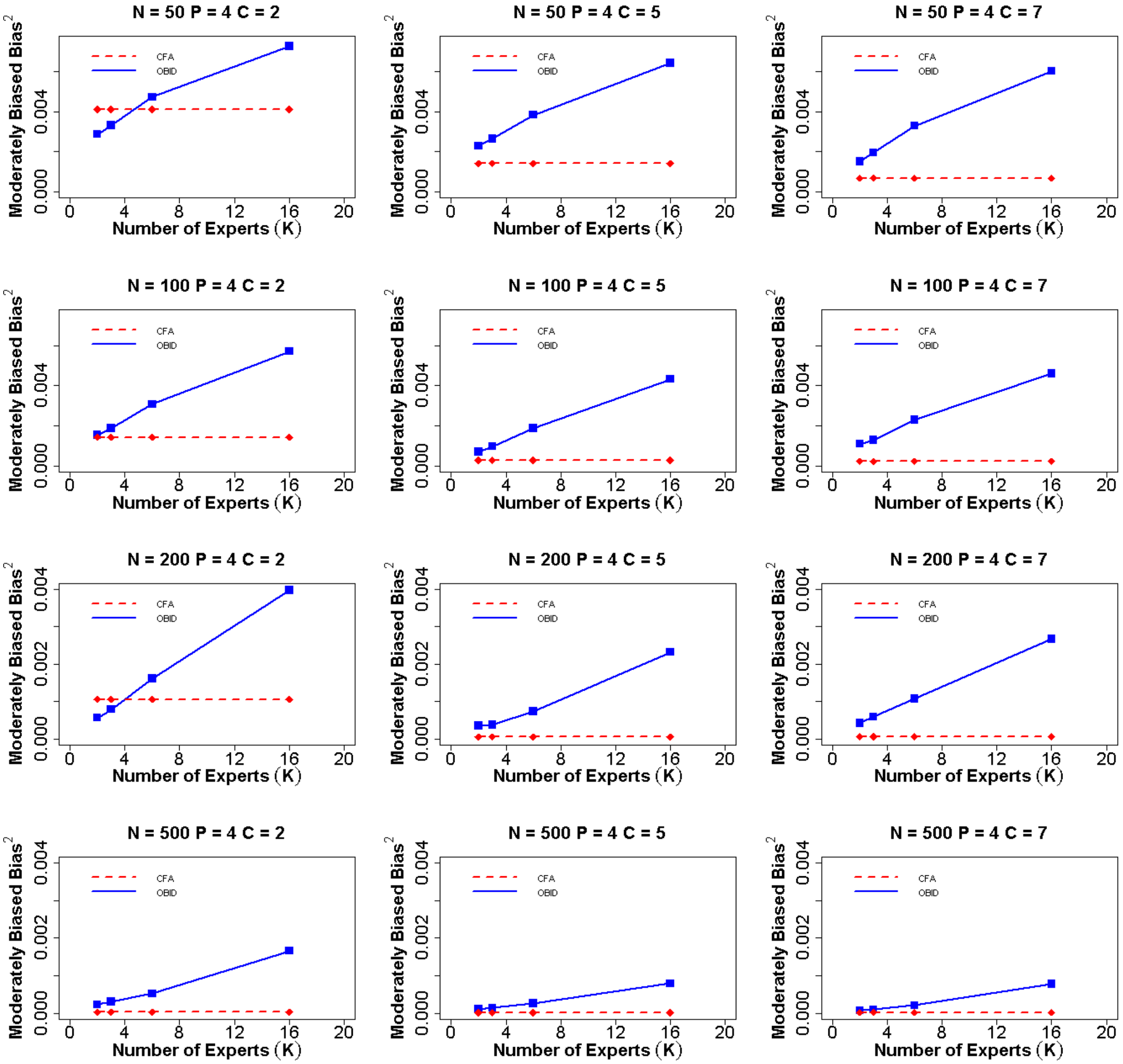

Figure 16. Average squared bias for item-to-domain correlation ρ using OBID (solid blue line) and ordinal CFA (dashed red line) when P = *4* (number of items) and experts are moderately biased $\{\rho_0 = (0.60, 0.40, 0.80, 0.60)\}$. The participant sample sizes are N = 50, 100, 200, and 500. The numbers of response categories are C = 2, 5, and 7, and the numbers of experts are K = 2, 3, 6, and 16.

Note. OBID = Ordinal Bayesian Instrument Development; CFA = Confirmatory Factor Analysis.

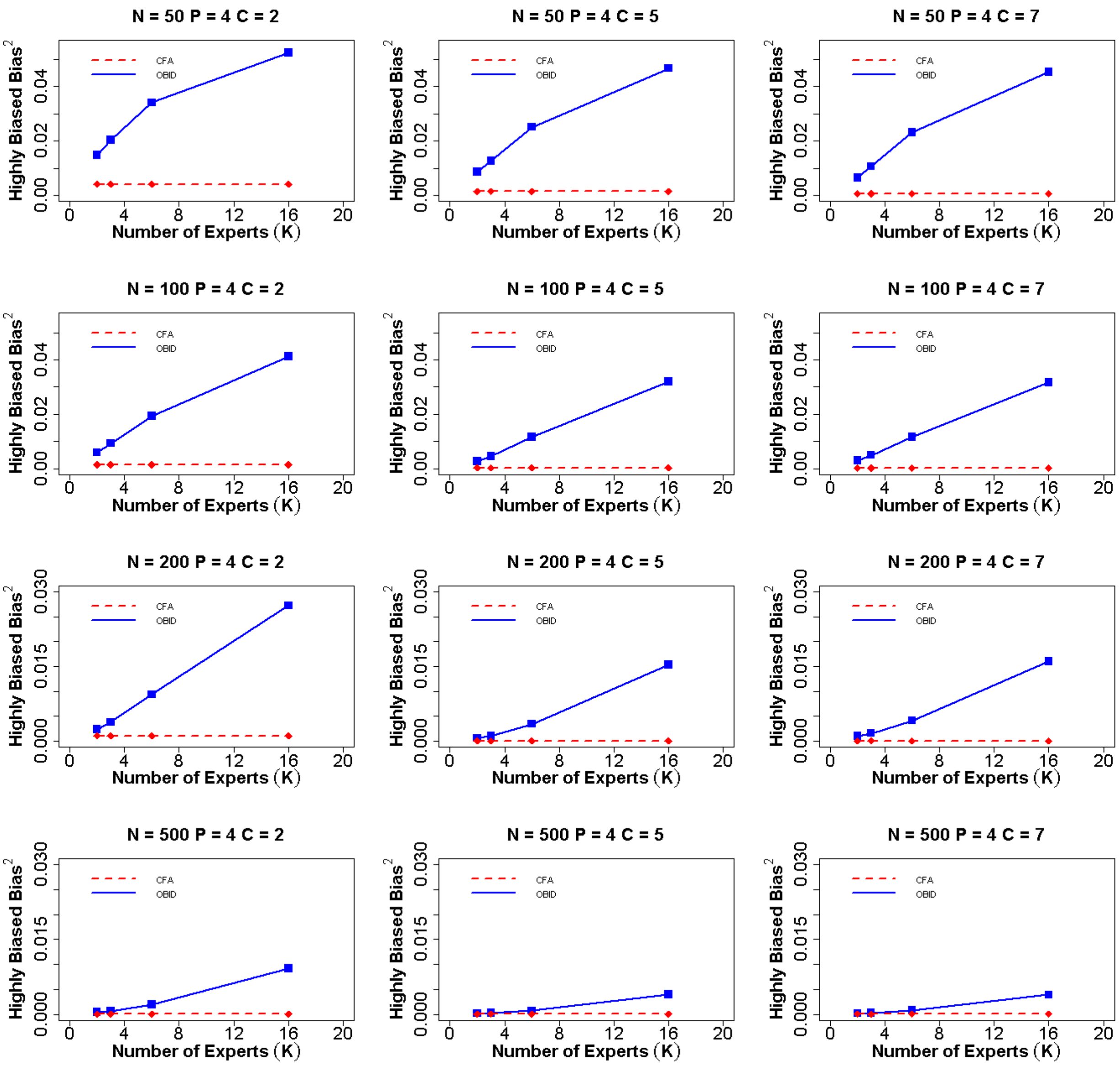

Figure 17. Average squared bias for item-to-domain correlation ρ using OBID (solid blue line) and ordinal CFA (dashed red line) when P = *4* (number of items) and experts are highly biased $\{\rho_0 = (0.75, 0.65, 0.85, 0.75)\}$. The participant sample sizes are N = 50, 100, 200, and 500. The numbers of response categories are C = 2, 5, and 7, and the numbers of experts are K = 2, 3, 6, and 16.

Note. OBID = Ordinal Bayesian Instrument Development; CFA = Confirmatory Factor Analysis.

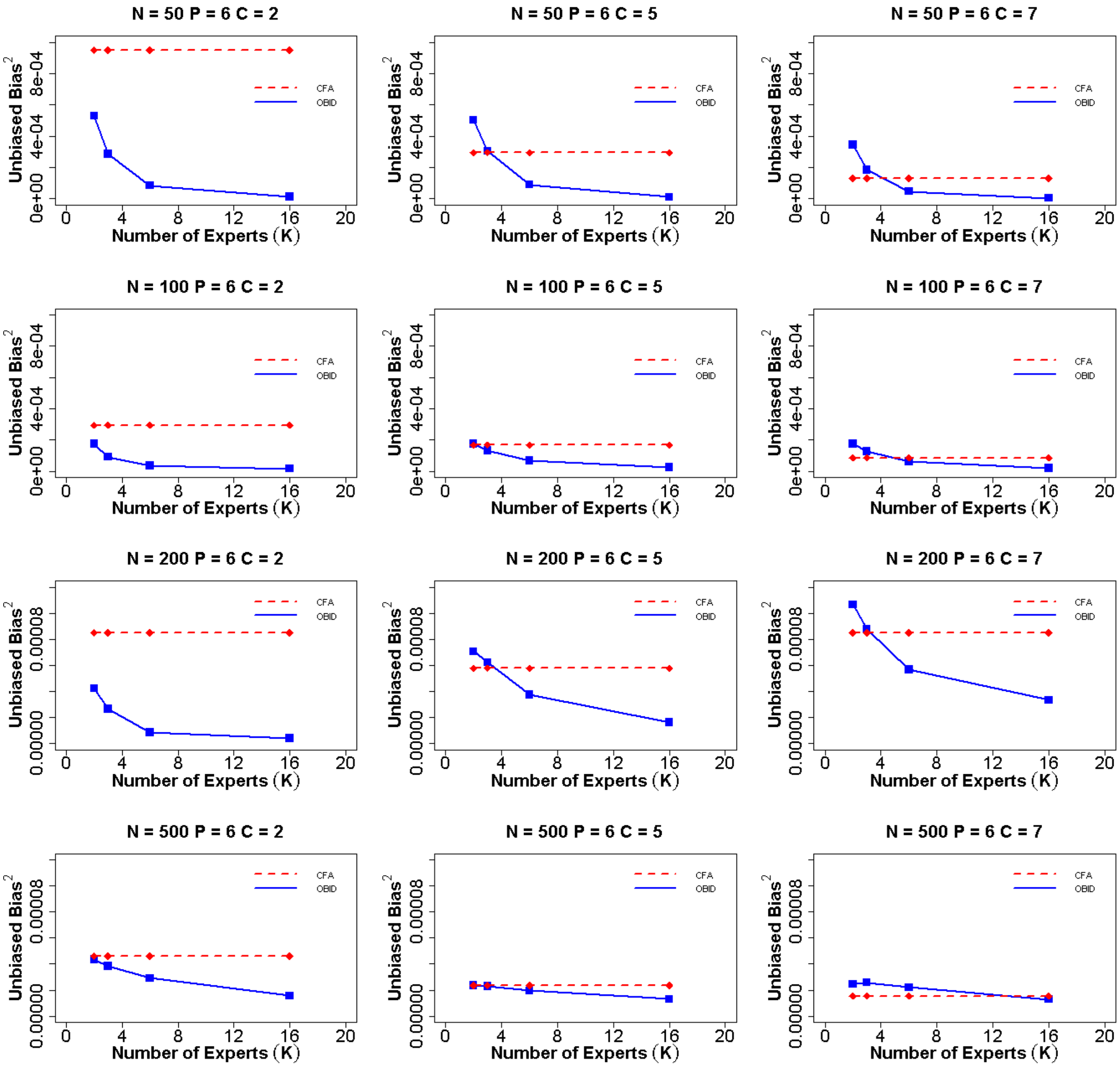

Figure 18. Average squared bias for item-to-domain correlation ρ using OBID (solid blue line) and ordinal CFA (dashed red line) when $P = 6$ (number of items) and experts are unbiased $\{\rho_0 = (0.30, 0.50, 0.70, 0.70, 0.30, 0.50)\}$. The participant sample sizes are N = 50, 100, 200, and 500. The numbers of response categories are C = 2, 5, and 7, and the numbers of experts are K = 2, 3, 6, and 16.

Note. OBID = Ordinal Bayesian Instrument Development; CFA = Confirmatory Factor Analysis.

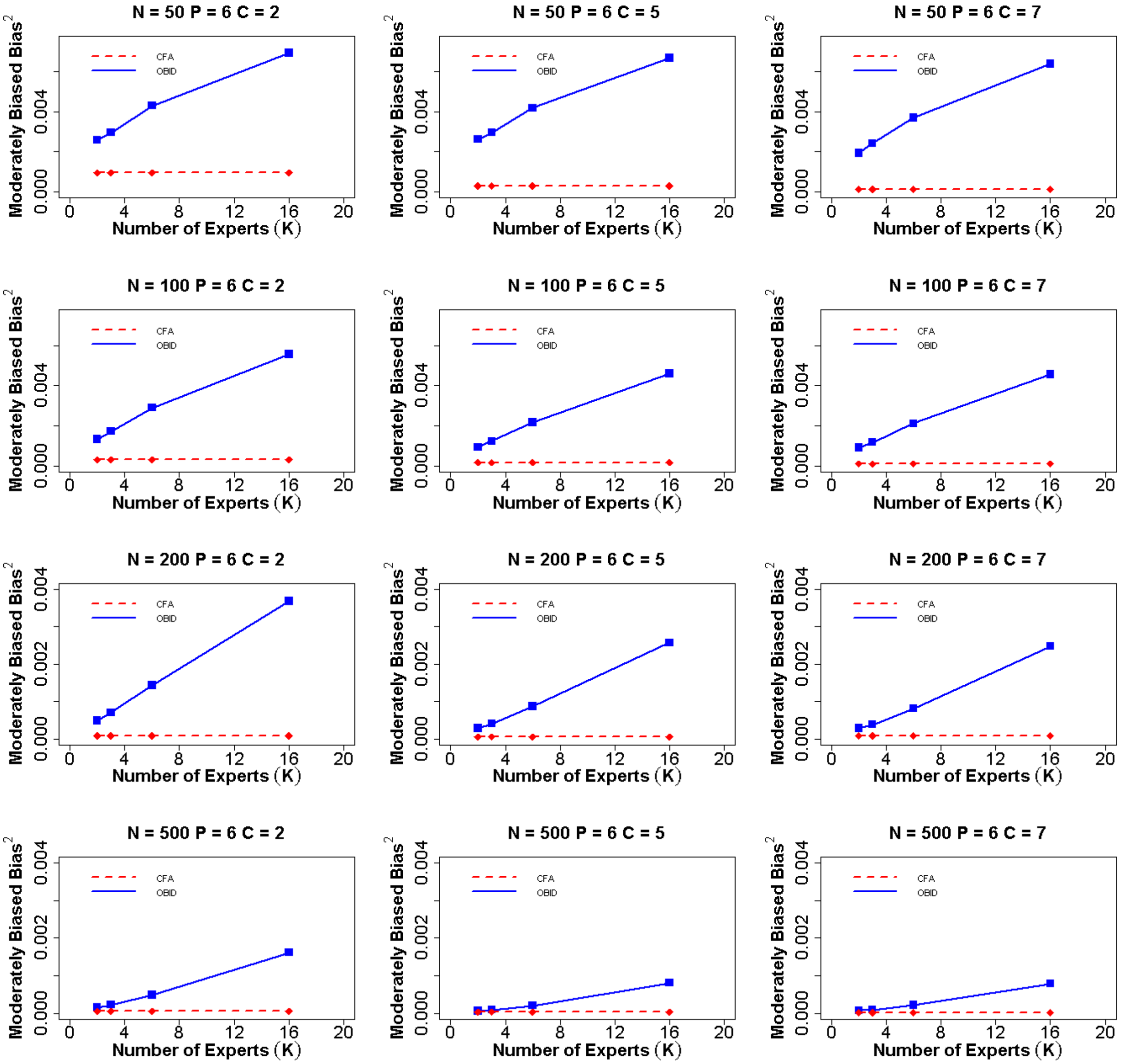

Figure 19. Average squared bias for item-to-domain correlation ρ using OBID (solid blue line) and ordinal CFA (dashed red line) when $P = 6$ (number of items) and experts are moderately biased $\{\rho_0 = (0.40, 0.60, 0.80, 0.80, 0.40, 0.60)\}$. The participant sample sizes are N = 50, 100, 200, and 500. The numbers of response categories are C = 2, 5, and 7, and the numbers of experts are K = 2, 3, 6, and 16.

Note. OBID = Ordinal Bayesian Instrument Development; CFA = Confirmatory Factor Analysis.

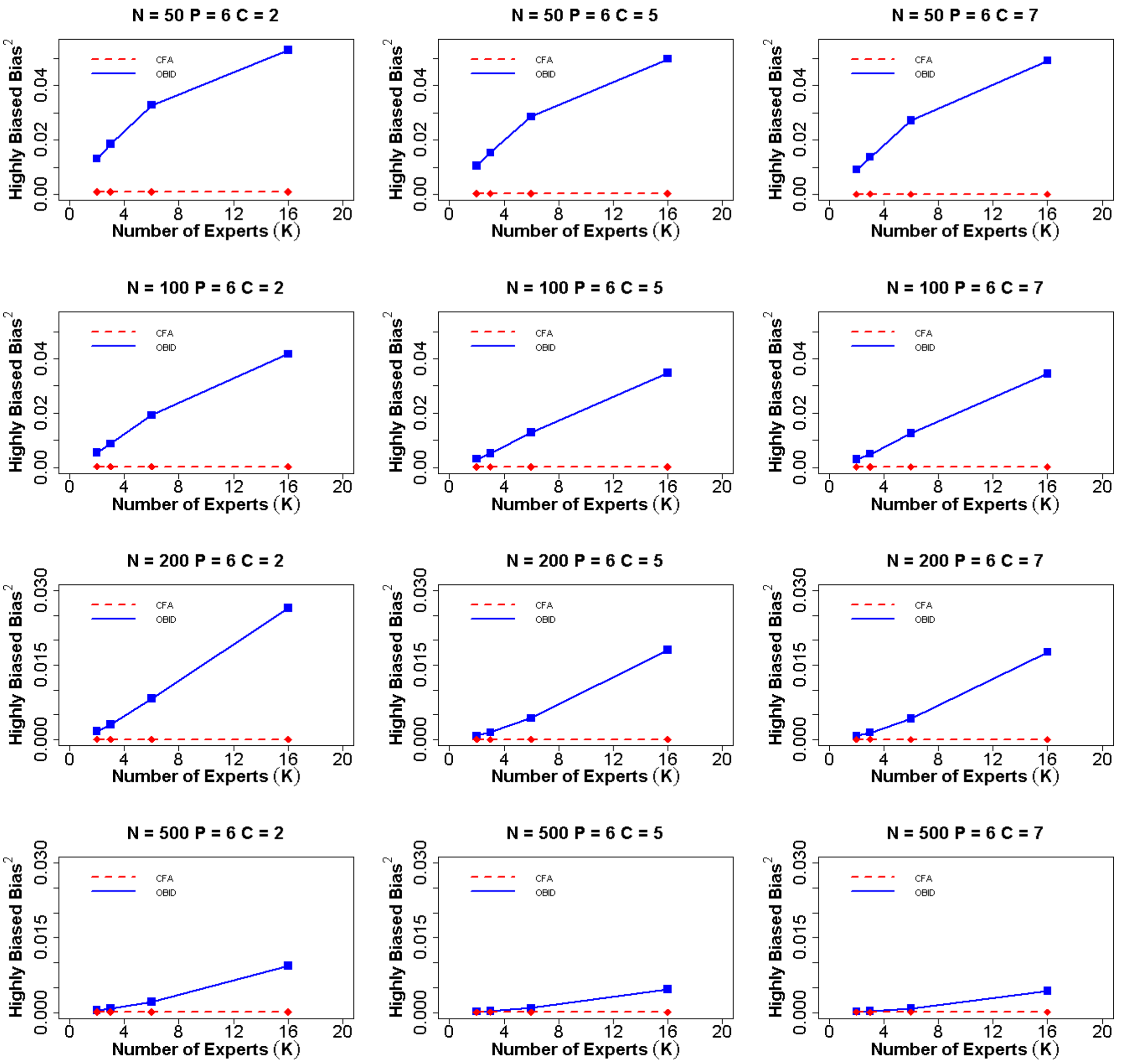

Figure 20. Average squared bias for item-to-domain correlation ρ using OBID (solid blue line) and ordinal CFA (dashed red line) when P = 6 (number of items) and experts are highly biased $\{\rho_0 = (0.65, 0.75, 0.85, 0.85, 0.65, 0.75)\}$. The participant sample sizes are N = 50, 100, 200, and 500. The numbers of response categories are C = 2, 5, and 7, and the numbers of experts are K = 2, 3, 6, and 16.

Note. OBID = Ordinal Bayesian Instrument Development; CFA = Confirmatory Factor Analysis.

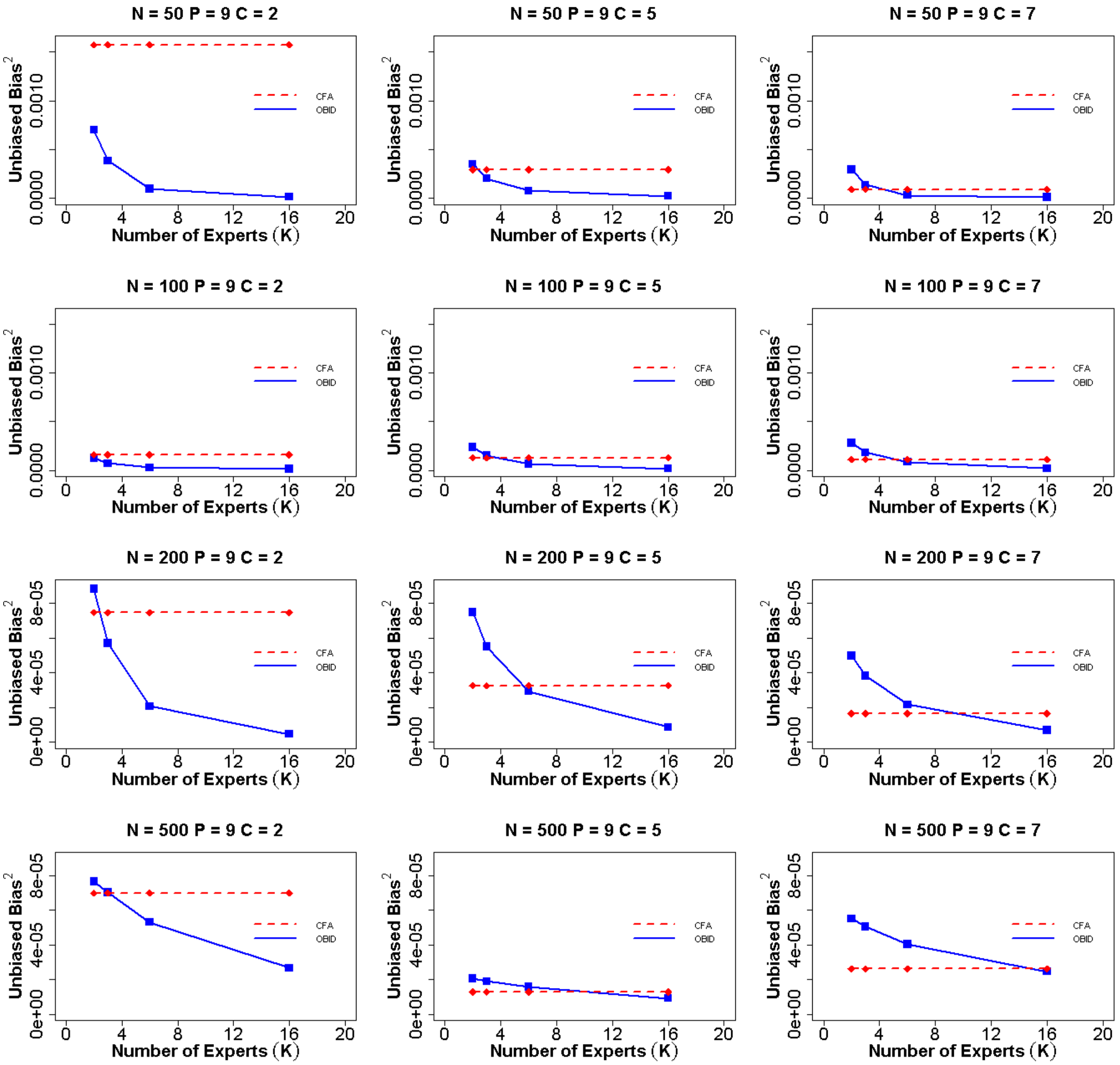

Figure 21. Average squared bias for item-to-domain correlation ρ using OBID (solid blue line) and ordinal CFA (dashed red line) when P = 9 (number of items) and experts are unbiased $\{\rho_0 = (0.30, 0.50, 0.70, 0.70, 0.30, 0.50, 0.70, 0.50, 0.30)\}$. The participant sample sizes are N = 50, 100, 200, and 500. The numbers of response categories are C = 2, 5, and 7, and the numbers of experts are K = 2, 3, 6, and 16.

Note. OBID = Ordinal Bayesian Instrument Development; CFA = Confirmatory Factor Analysis.

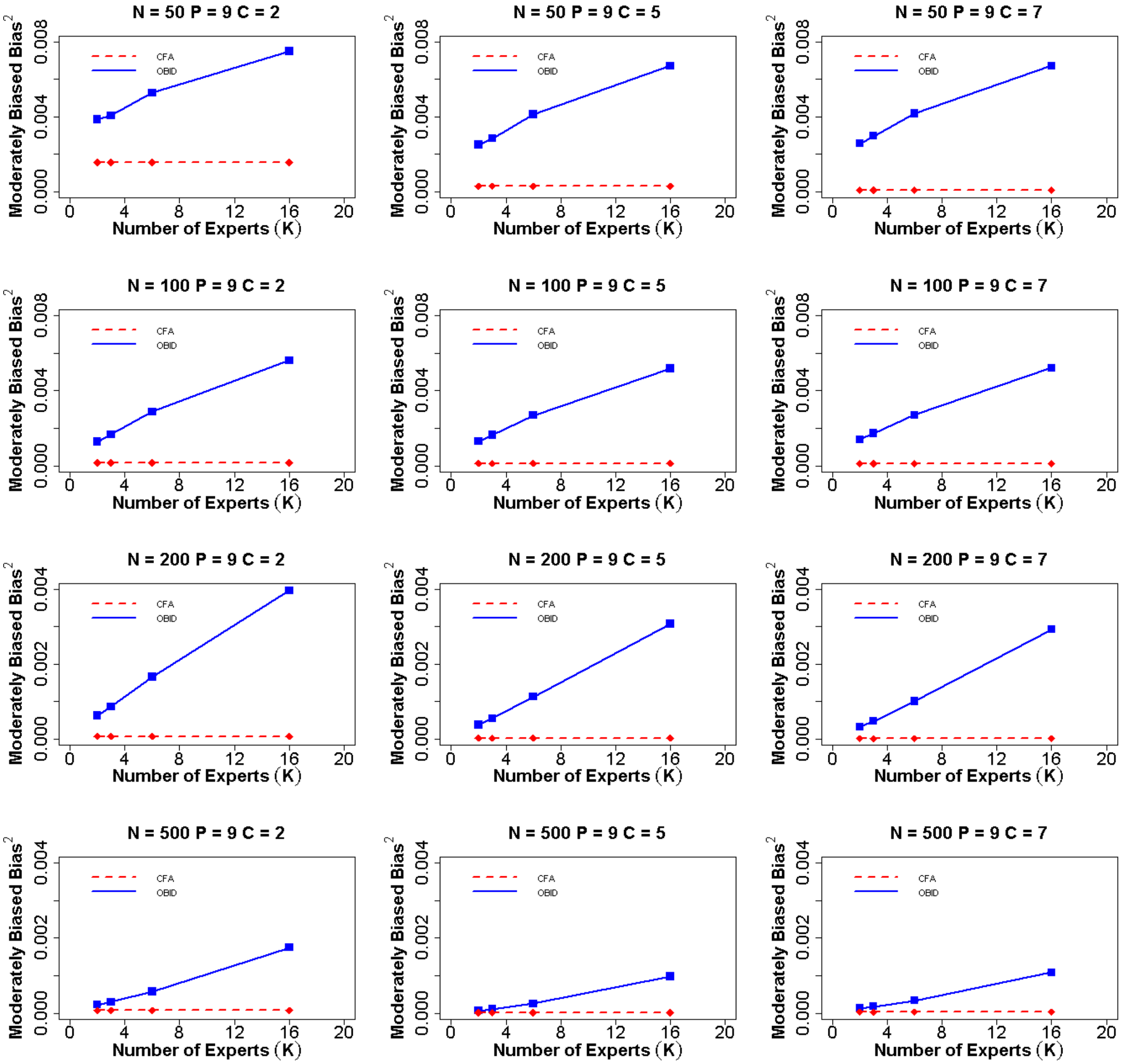

Figure 22. Average squared bias for item-to-domain correlation ρ using OBID (solid blue line) and ordinal CFA (dashed red line) when $P = 9$ (number of items) and experts are moderately biased $\{\rho_0 = (0.40, 0.60, 0.80, 0.80, 0.40, 0.60, 0.80, 0.60, 0.40)\}$. The participant sample sizes are N = 50, 100, 200, and 500. The numbers of response categories are C = 2, 5, and 7, and the numbers of experts are K = 2, 3, 6, and 16.

Note. OBID = Ordinal Bayesian Instrument Development; CFA = Confirmatory Factor Analysis.

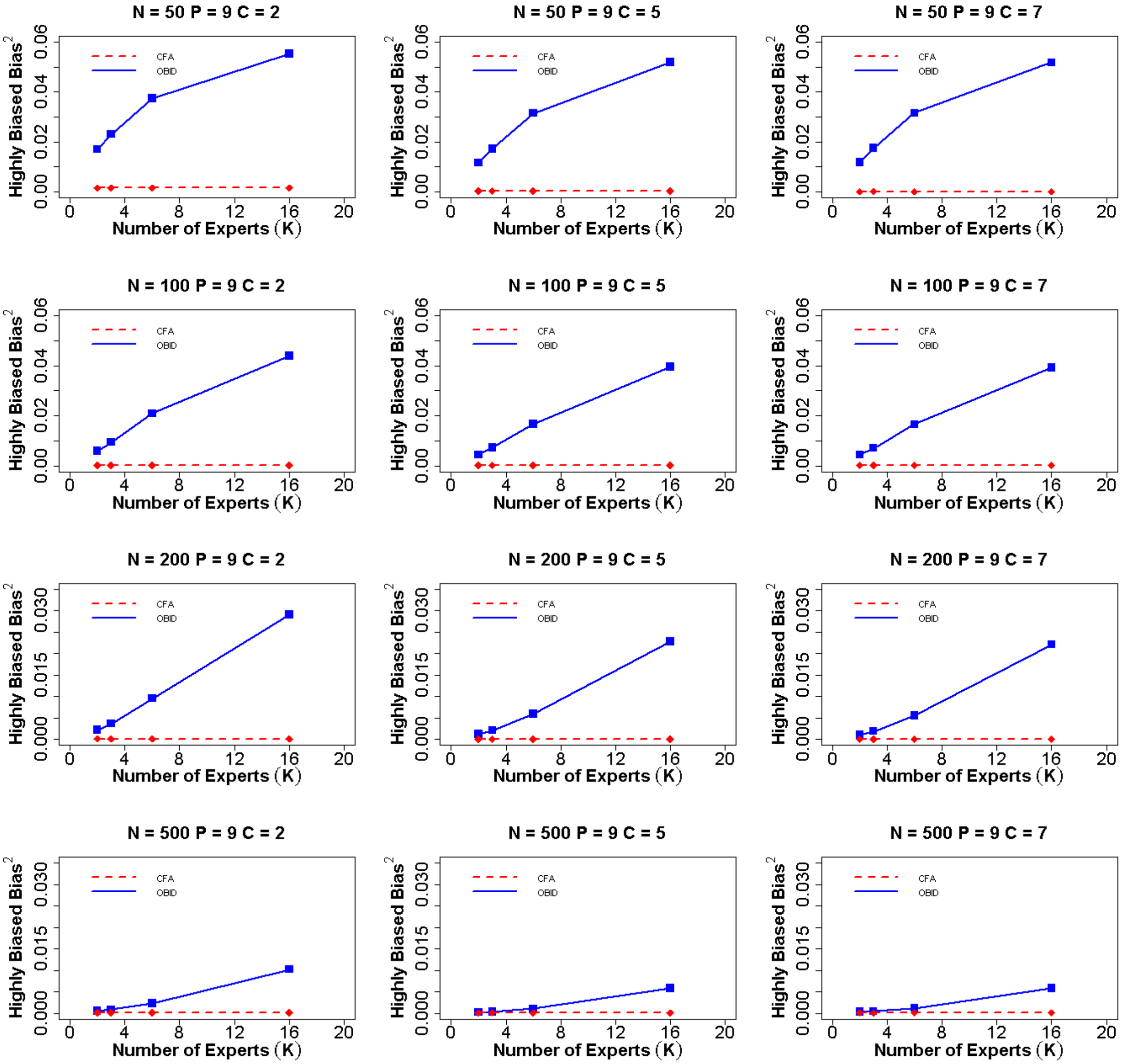

Figure 23. Average squared bias for item-to-domain correlation ρ using OBID (solid blue line) and ordinal CFA (dashed red line) when P = *9* (number of items) and experts are highly biased $\{\rho_0 = (0.65,0.75,0.85,0.85,0.65,0.75,0.85,0.75,0.65)\}$. The participant sample sizes are N = 50, 100, 200, and 500. The numbers of response categories are C = 2, 5, and 7, and the numbers of experts are K = 2, 3, 6, and 16.

Note. OBID = Ordinal Bayesian Instrument Development; CFA = Confirmatory Factor Analysis.
